# Ricci Curvature Tensor-Based Volumetric Segmentation

**DOI:** 10.1007/s11263-025-02492-6

**Published:** 2025-06-15

**Authors:** Jisui Huang, Ke Chen, Andreas Alpers, Na Lei

**Affiliations:** 1 School of Mathematical Sciences, Capital Normal University, West Third Ring Road North, Haidian District, 100048 Beijing China; 2 Centre for Mathematical Imaging Techniques and Department of Mathematical Sciences, University of Liverpool, Peach Street, Liverpool, L697ZL Merseyside United Kingdom; 3 Department of Mathematics and Statistics, University of Strathclyde, Richmond St, Glasgow, G11XH Scotland United Kingdom; 4 School of Software, Dalian University of Technology, Linggong Road, Dalian, 116024 Liaoning China

**Keywords:** Riemannian geometry, Ricci curvature tensor, Image segmentation, Variational model

## Abstract

Existing level set models employ regularization based only on gradient information, 1D curvature or 2D curvature. For 3D image segmentation, however, an appropriate curvature-based regularization should involve a well-defined 3D curvature energy. This is the first paper to introduce a regularization energy that incorporates 3D scalar curvature for 3D image segmentation, inspired by the Einstein-Hilbert functional. To derive its Euler-Lagrange equation, we employ a two-step gradient descent strategy, alternately updating the level set function and its gradient. The paper also establishes the existence and uniqueness of the viscosity solution for the proposed model. Experimental results demonstrate that our proposed model outperforms other state-of-the-art models in 3D image segmentation.

## Introduction

Image segmentation is an essential task in image processing that aims at partitioning an image into semantically meaningful classes. It facilitates a subsequent image analysis and has found various applications, e.g., in object identification and recognition, volume rendering, and lesion localization (Lei & Nandi, 2022).

Among the various segmentation techniques, level set methods (Wang et al., 2021; Yu et al., 2020; Biswas & Hazra, 2022; Kang et al., 2019) have gained considerable attention due to their robustness and versatility. These methods, based on curve evolution theory, provide a powerful framework for outlining complex structures within images. Typically, level set methods employ a cost function that consists of two terms: a region term and an edge term.

The region term guides data fidelity by approximation, splitting the image into homogeneous groups of voxels. A prominent example is given by the renowned Chan-Vese model (Chan & Vese, 2001), which assumes the foreground and the background have respectively uniform intensities. To adapt to complex real datasets, the model was extended to images with heterogeneous regions using local regional information. Examples include local statistical information (Wang et al., 2010), weighted region information (Li et al., 2007) calculated using a Gaussian kernel-based convolution, geodesic distance between two distinct spectral density functions (Li et al., 2017), and geodesic distance between two fitted Gaussian distributions (Lenglet et al., 2005).

Region terms, however, can be sensitive to initialization. An effective approach to overcome this problem is to incorporate prior information into the region term by employing a selective model. In the selective model, contour evolution is driven by the force generated from user-specified points. Nguyen et al. (2012) introduced an additional force to the level set originating from points in- and outside the object of interest. Gout et al. (2005) employed the prior boundary information to encourage the level set to pass along these pre-defined points. Spencer and Chen (2015) replaced the distance term in the edge term of the Gout et al. model by a stand-alone term. Additionally, the authors introduced a numerically stable, convex relaxation of their model (we refer to it as relaxed level set) by replacing the discrete values of 0 and 1 from the Heaviside function by a smooth function which gradually increases from 0 to 1. However, their model, employing the Euclidean distance to marker voxels as the distance measure, cannot incorporate image color (or grayscale) information. To address this, Roberts et al. (2019) replaced the Euclidean distance by the geodesic distance, which does not only take spatial variances but also grayscale variances into account.

The edge term, on the other hand, encourages the level set to stop at image boundaries. A fundamental edge-based level set model is the geodesic active contour (Caselles et al., 1997), which integrates the gradients of the level set and image. The contour, therefore, balances between passing along edges and remaining smooth. To improve the efficiency, the DRLSE model (Li et al., 2010) introduced a new energy term to eliminate the need for reinitialization. Su et al. (2020) extracted different gradient-based edge information from images of various resolutions. To mitigate the adverse effect of noise, several works combined gradient information with other forms of information. For example, Yu et al. (2019) incorporated the diffusion rate to the edge term. Liu et al. (2017) incorporated the local regional fitting variances to the gradient information, which was then developed into the multi-local statistical information (Liu et al., 2019).

Edge descriptors based on gradients, however, are less effective for images with high noise or low contrast. Recently, a rapidly growing body of literature has emerged in which some notion of curvature is incorporated into the edge term. Based on the dimension, one can classify these approaches into 1D and 2D curvature approaches. A well-known 1D curvature is the Euler’s Elastica. Zhu et al. (2013) proposed a model that uses Euler’s Elastica, a function of the mean curvature, as a regularizer. As the resulting Euler-Lagrange equation is a complex fourth-order equation, the authors introduced a variety of auxiliary variables to break down the original problem into different subproblems. In He et al. (2019), the $\ell ^{1}$ norm of Euler’s Elastica is discussed. Zhong et al. (2020) proposed a different function of the mean curvature as the regularization. 2D curvature is also widely used in image processing. Liu et al. (2022) proposed a Gaussian curvature-based regularization for image denoising and surface smoothing. Zhu and Chan (2012) used the mean curvature of the surface determined by the image to implement image denoising.

**Fig. 1:**
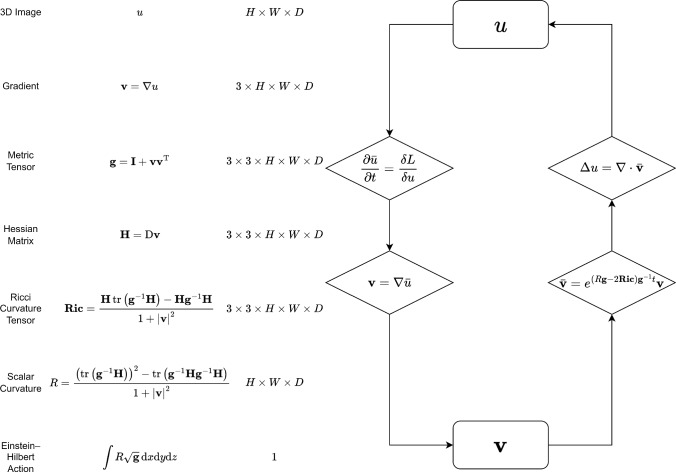
Overall notation (left) and pipeline (right) of our model. The first two columns show names and formulas, including the, for our approach, relevant 3D intrinsic curvatures; here ${\textbf{I}}$ and $\operatorname {D}$ denote the identity matrix and the Jacobian matrix of the map ${\textbf{v}},$ respectively. The third column provides their respective shapes with *H*, *W*, and *D* denoting the image height, width, and depth, respectively. What prevents a wide application of some curvature flow is known to lie in a dilemma: is it possible to embed a manifold into the Euclidean space using metric tensor ${\textbf{g}}$ (3), which has proved to be independent of ambient space? This paper attempts to avoid this problem by incorporating the embedding information into the Euler-Lagrange equation by performing variational calculus with respect to the gradient rather than the metric tensor. For an arbitrary 3D function (e.g., a 3D image or a relaxed level set), we alternately update the original function and its gradient, as shown graphically on the right-hand side of this figure. An implication of this new approach is the prospect of applying a wide range of intrinsic curvature flows, such as the renowned Ricci flow, to the field of image processing

However, rather than being functions implicitly representing 2D surfaces, relaxed level set functions are genuinely 3D functions. It would be more powerful to directly optimize the 3D manifold itself rather than optimizing 2D submanifolds. To the best of our knowledge, the use of 3D curvature as a regularization has not been investigated. Three challenges need to be overcome. The first is to define a proper energy function that can smooth the 3D manifold determined by the 3D level set. The second is to derive a compact formula of the curvature energy. The third is to derive its Euler-Lagrange equation, which is perhaps the most essential and technically most complex of the three.

In this paper, we present a novel model that utilizes curvature as regularization. Drawing inspiration from the Einstein-Hilbert functional (Feynman, 2018), we propose the regularization

1 $$\begin{aligned} \int R(u(x,\,y,\,z))\,\textrm{d}\mu , \end{aligned}$$

where $u(x,\,y,\,z)$ represents a 3D function (e.g., a 3D medical image or a user-defined 3D function), *R* denotes the scalar curvature of the three-dimensional manifold $(x,\,y,\,z,\,u(x,\,y,\,z))$, and $\textrm{d}\mu$ denotes the volume element of this manifold. The scalar curvature *R* and the volume element $\textrm{d}\mu$ are uniquely determined by the metric tensor ${\textbf{g}}$, a $3\times 3$ positive definite matrix assigned to each point of the manifold. The metric tensor, which can be used, e.g., to define the pairwise geodesic distances between points on the manifold, is uniquely determined by the intrinsic nature of the manifold (Do Carmo & Flaherty Francis, 1992). Specifically, ${\textbf{g}}$ is invariant to transformations (including rotations and translations) of the coordinate system, and it is invariant to its embedding into an ambient space (e.g., an Euclidean or hyperbolic space). This property allows to define an intrinsic Eulerian-Lagrangian equation with respect to, instead of the original 3D parametric surface $(x,\,y,\,z,\,u(x,\,y,\,z))$, the metric tensor ${\textbf{g}}$, as is often the case in variational calculus in Riemannian geometry. Consequently, the Eulerian-Lagrangian of the Einstein-Hilbert functional is also a function of the metric tensor ${\textbf{g}}$, known as the Einstein tensor (Einstein et al., 1916).

However, this appealing intrinsic property limits its broad application to image processing. The independence of coordinate systems and ambient space implies that we cannot determine how to embed the new manifold with the updated metric tensor ${\textbf{g}}$ into the Euclidean space, or even the dimension in which the new manifold should reside. In other words, although we derive the metric tensor of the specific parametric hypersurface $(x,\,y,\,z,\,u(x,\,y,\,z))$ in the 4D Euclidean space, the new parametric surface may not be parameterizable in the same form, or the new surface might only exist in a 5D space.

A potential way to deal with this is to restrict the metric’s transformation to a particular form. In 2D cases, the discrete Ricci flow (Gu & Yau, 2016) only allows the metric tensor to undergo a conformal transformation. According to the uniformization theorem (Abikoff, 1981), a mesh with a genus greater than one is conformally equivalent to the Poincaré Hyperbolic Disk. Thus, after updating the metric tensor, we can combine a variety of triangles with known lengths into a unit disk using the law of cosines (Gu & Yau, 2016). However, it is not understood whether or not this can be extended to 3D cases.

To address this, we restrict the manifold to a particular form, namely, the hypersurface in a 4D Euclidean space, which is a natural setting for 3D image processing. At this point, the metric tensor can be determined by the image gradient, which allows us to directly derive the variational calculus with respect to the image gradient, rather than the metric tensor, in Euclidean coordinates. Before proceeding with segmentation, we first lay some foundation work. We derive the explicit formulas for the metric tensor ${\textbf{g}}$, the Ricci curvature tensor $\textbf{Ric}$, and the scalar curvature *R* of the 3D manifold $(x,\,y,\,z,\,u(x,\,y,\,z))$, as shown in Figure 1. Next, we derive the variational calculus with respect to the gradient $\nabla u$, leading to a two-stage strategy to update our model. We alternately update *u* and $\nabla u$, in contrast to the numerous additional auxiliary variables introduced by variable splitting, as used by Zhong et al. (2020) to optimize the energy involving Euler’s Elastica.

Our contributions can be summarized as follows:

1. This is the first study to introduce a regularization incorporating 3D scalar curvature into 3D image segmentation.
2. We implement a two-step update strategy to update the level set function and its gradient alternately.
3. We prove the existence and uniqueness of the viscosity solution.
4. Experiments show that our model can outperform other state-of-the-art models.

The paper is structured as follows. In Section 2, we briefly review some prior selective variational models. In Section 3, we introduce our proposed level set model that incorporates the Einstein-Hilbert action, including the definition of the Einstein-Hilbert action for the 3D Riemannian manifold determined by a 3D image, and the derivation of its Euler-Lagrange equation. In Section 4, we discuss the existence and uniqueness of the viscosity solution. In Section 5, we conduct a comprehensive evaluation on real datasets and compare our method with the state-of-the-art model. Finally, we conclude in Section 6.

## Review of Selective Variational Models

Given a 3D image $f(x,\,y,\,z)$, a level set model aims to find a level set function $u(x,\,y,\,z)$ that minimizes a prescribed functional. Variational models can be classified into global variational models and selective variational models, depending on whether or not user interaction is required. In this paper, we focus on the selective model, and thus, we first briefly review some related selective variational models.

### Nguyen Model

The Nguyen model (Nguyen et al., 2012) identifies a relaxed function $u(x,\,y,\,z)$ by exploiting the following energy

$$\begin{aligned}&\gamma \int \beta h\left( P_F\left( x,\,y,\,z\right) \right) |\nabla u|\,\textrm{d}\Omega \\&\quad +\gamma \int \left( 1-\beta \right) h\left( f\right) |\nabla u|\,\textrm{d}\Omega \\&\quad +\lambda \int \alpha \left( P_B\left( x,\,y,\,z\right) -P_F\left( x,\,y,\,z\right) \right) u\,\textrm{d}\Omega \\&\quad +\lambda \int \left( 1-\alpha \right) \left( 1-2P\left( x,\,y,\,z\right) \right) u\,\textrm{d}\Omega , \end{aligned}$$

where $h\left( f\right) =\frac{1}{1+{|\nabla f|}^2}$ is the edge descriptor, which encourages the level set to follow along edges. As usual, $\alpha$, $\beta$, $\gamma$, and $\lambda$ are regularization parameters. The model combines two aspects. On the one hand, a Gaussian mixture model estimated from the foreground and background seeds is used to derive the likelihoods $P_F(x,y,z)$ and $P_B(x,y,z),$ that a voxel belongs to the foreground and background, respectively. On the other hand, a normalized geodesic distance function $P\left( x,\,y,\,z\right)$ is incorporated, which measures the distance of a voxel to an already segmented foreground region.

### Spencer-Chen Model

The Spencer-Chen model (Spencer & Chen, 2015) employs the energy functional

$$\begin{aligned} \begin{aligned}&\int \left( \lambda _1{\left( f-c_1\right) }^2 - \lambda _2{\left( f-c_2\right) }^2\right) u\,\textrm{d}\Omega \\&\quad + \int \gamma h\left( f\right) |\nabla u|\,\textrm{d}\Omega +\theta {\mathcal {D}}_E\left( x,\,y,\,z\right) u\,\textrm{d}\Omega \\&\quad + \alpha \int \nu \left( u\right) \,\textrm{d}\Omega , \end{aligned} \end{aligned}$$

where ${\mathcal {D}}_E(x,\,y,\,z)$ measures the Euclidean distance between the point (*x*, *y*, *z*) and user-specified foreground seeds, $\nu :{\mathbb {R}}\mapsto {\mathbb {R}}$ denotes a well-designed function enforcing $u(x,\,y,\,z)$ to lie in the range $[0,\,1]$, and $\alpha ,$ $\gamma ,$ $\lambda _1$, $\lambda _2$, $\theta ,$ $c_1$, $c_2$, are scalar parameters. Among these parameters, $c_1$ and $c_2$ can be modified during each iteration, while the others remain constant. When fixing $u(x,\,y,\,z)$, we can derive the optimized $c_1$ and $c_2$:

$$\begin{aligned} c_1&=\frac{\int u(x,\,y,\,z)f(x,\,y,\,z)\,\textrm{d}\Omega }{\int u(x,\,y,\,z)\,\textrm{d}\Omega }\\ c_2&=\frac{\int \left( 1-u\left( x,\,y,\,z\right) \right) f\left( x,\,y,\,z\right) \,\textrm{d}\Omega }{\int \left( 1-u(x,\,y,\,z)\right) \,\textrm{d}\Omega }. \end{aligned}$$

### Roberts-Chen Model

The Roberts-Chen model (Roberts et al., 2019), originally formulated in two-dimensional space, employs a similar energy as the Spencer-Chen model. The difference is that ${\mathcal {D}}_E$ is replaced by ${\mathcal {D}}_G,$ with ${\mathcal {D}}_G(x,\,y,\,z)$ measuring the geodesic distance between the point (*x*, *y*, *z*) and user-specified foreground seeds.

### Other Models

Some models do not directly use level sets for segmenting a given image. For example, the weighted variational model in Liu et al. (2018) focuses on the level set function fitting the original image as accurately as possible, followed by image thresholding.

Duan et al. (2014) utilize two convex variational models: one is a smoothed image, and the other one performs clustering. However, these models feature a common term $\int h|\nabla u| \textrm{d}\Omega$, which is a weighted form of the gradient-based regularization $\int |\nabla u|\,\textrm{d}\Omega$. As shown in the 2D image segmentation, existing 1D and 2D curvature-based regularization, such as Euler’s Elastica (Duan et al., 2014), can outperform the traditional gradient-based term.

## Ricci Curvature Based Model

In this section, we introduce our proposed selective variational model for 3D image segmentation, which incorporates the Einstein-Hilbert action as an additional regularization term. To formulate the Einstein-Hilbert action, we first provide explicit formulas for various curvatures of the 3D Riemannian manifold $(x,\,y,\,z,\,u(x,\,y,\,z))$, corresponding to a 3D function $u(x,\,y,\,z)$. These include the Riemann curvature tensor, the Ricci curvature tensor, and the scalar curvature, as will be detailed in Section 3.1. Utilizing the explicit formulas, we present our selective variational model in Section 3.2. The regularization in this model encourages the level set function to resemble the smoothest possible manifold, namely, the Euclidean space. However, in Riemannian geometry, curvature tensors are functions of the metric tensor, which poses a challenge in possible updating strategies for *u*. To address this, we reformulate the Einstein-Hilbert action as a function of the gradient. Consequently, our proposed level set model has two arguments: the original level set and its gradient. In Section 3.3, we derive the Euler-Lagrange equation with respect to these two variables separately, followed by a discussion of implementation details in Section 3.4.

### Einstein-Hilbert Action

In this section, we shall show that the Einstein-Hilbert action (1), a functional mapping the 3D function $u(x,\,y,\,z)$ to a scalar, can be expressed as

2 $$\begin{aligned} \begin{aligned}&\int R(u(x,\,y,\,z))\,\textrm{d}\mu \\&\quad =\int \frac{{\left( \operatorname {tr}\left( {\textbf{g}}^{-1}{\textbf{H}}\right) \right) }^2-\operatorname {tr}\left( {\textbf{g}}^{-1}{\textbf{H}}{\textbf{g}}^{-1}{\textbf{H}}\right) }{\sqrt{1+{|\nabla u|}^2}}\,\textrm{d}x\textrm{d}y\textrm{d}z. \end{aligned} \end{aligned}$$

To this end, we consider $u(x,\,y,\,z)$ being represented as the three-dimensional parametric surface ${\textbf{r}}\left( x,\,y,\,z\right) =(x,\,y,\,z,\,u(x,\,y,\,z))$ in four-dimensional Euclidean space. Recall that *R* denotes the scalar curvature of the manifold ${\textbf{r}}\left( x,\,y,\,z\right)$, and $d\mu$ denotes its volume element. In the following, we will derive the right-hand side of (2) by combining formulas for the metric tensor, the Riemann curvature tensor, and the Ricci curvature tensor. We will introduce the relevant notation as we proceed.

For each point of ${\textbf{r}}\left( x,\,y,\,z\right) ,$ we have the tangents ${\textbf{r}}_1=(1,\,0,\,0,\,u_x)$, ${\textbf{r}}_2=(0,\,1,\,0,\,u_y),$ and ${\textbf{r}}_3=(0,\,0,\,1,\,u_z)$. The metric tensor ${\textbf{g}},$ which is a $3\times 3$ matrix with components $g_{ij}$ given by the pair-wise inner products $g_{ij}=<{\textbf{r}}_i,{\textbf{r}}_j>,$ can therefore be expressed as

3 $$\begin{aligned} {\textbf{g}}={\textbf{I}}+\nabla u {\nabla u}^{\operatorname {T}}, \end{aligned}$$

where ${\textbf{I}}$ denotes the identity matrix.

The Riemann curvature tensor $R_{ijkl},$ a $3\times 3\times 3\times 3$ tensor, can be expressed (see (A3)) as

4 $$\begin{aligned} R_{ikjl} =\frac{u_{ij}u_{kl}-u_{il}u_{jk}}{1+{|\nabla u|}^2}, \end{aligned}$$

with $u_{ij}$ denoting the components of the Hessian matrix ${\textbf{H}}$ of *u*.

The Ricci curvature tensor $\textbf{Ric},$ a $3\times 3$ tensor with components denoted by $R_{ij},$ and the Riemann curvature tensor are related (see, e.g., Petersen (2016a)) by

5 $$\begin{aligned} R_{ij}=g^{kl}R_{ikjl}; \end{aligned}$$

here $g^{kl}$ denotes the *k*th row and *l*th column entry of the inverse matrix ${\textbf{g}}^{-1},$ and we are following the Einstein summation convention, where we automatically sum over indices that are repeated as both subscripts and superscripts.

As $\operatorname {tr}({\textbf{g}}^{-1}{\textbf{H}})=g^{kl}u_{kl}$ and ${\textbf{H}}{\textbf{g}}^{-1}{\textbf{H}}=u_{il}g^{lk}u_{kj}$, we therefore obtain in matrix notation

6 $$\begin{aligned} \textbf{Ric}=\frac{{\textbf{H}}\operatorname {tr}\left( {\textbf{g}}^{-1}{\textbf{H}}\right) -{\textbf{H}}{\textbf{g}}^{-1}{\textbf{H}}}{1+{|\nabla u|}^2}. \end{aligned}$$

The Ricci curvature tensor and the scalar curvature *R* are related via

7 $$\begin{aligned} R=\operatorname {tr}\left( {\textbf{g}}^{-1}\textbf{Ric}\right) ; \end{aligned}$$

see, e.g., Petersen (2016a). Hence, by substituting (6) into (7), we obtain

8 $$\begin{aligned} \begin{aligned} R=\frac{{\left( \operatorname {tr}\left( {\textbf{g}}^{-1}{\textbf{H}}\right) \right) }^2-\operatorname {tr}\left( {\textbf{g}}^{-1}{\textbf{H}}{\textbf{g}}^{-1}{\textbf{H}}\right) }{1+{|\nabla u|}^2}. \end{aligned} \end{aligned}$$

This, together with the definition of the volume element (see e.g., Petersen (2016b)),

$$\begin{aligned} \textrm{d}\mu =\sqrt{ \det \left( {\textbf{g}}\right) }\,\textrm{d}x\textrm{d}y\textrm{d}z \end{aligned}$$

and the well-known matrix identity

$$\begin{aligned} \det \left( {\textbf{I}}+{\textbf{u}}{\textbf{v}}^{\operatorname {T}}\right) =1+{\textbf{u}}^{\operatorname {T}}{\textbf{v}} \end{aligned}$$

yields (2).

An interesting property of the Einstein-Hilbert functional is its invariance under coordinate transformations (see Appendix B). In addition, it has been proved that the functional is invariant under local isometries, which, originally for 2D manifolds, is due to K. F. Gauss (Pressley, 2010). This invariance implies that if the solution *u* of the variational calculus of the functional undergoes an isometric transformation (e.g., a translation or rotation), it remains to be a solution.

### The Proposed Model

Drawing inspiration from the Roberts-Chen model (Section 2.3), we introduce our proposed selective variational model for a given three-dimensional image $f(x,\,y,\,z)$ as follows:

9 $$\begin{aligned} \begin{aligned}&\min _u\int \lambda _1 {\left( f(x,\,y,\,z)-c_1\right) }^2u\left( x,\,y,\,z\right) \,\textrm{d}\Omega \\&\quad -\int \lambda _2{\left( f(x,y,z)-c_2\right) }^2u\left( x,\,y,\,z\right) \,\textrm{d}\Omega \\&\quad +\theta \int {\mathcal {D}}_G\left( x,\,y,\,z\right) u\left( x,\,y,\,z\right) \,\textrm{d}\Omega \\&\quad + \alpha \int \nu _\epsilon \left( u\left( x,\,y,\,z\right) \right) \,\textrm{d}\Omega \\&\quad + \int R\left( u\left( x,\,y,\,z\right) \right) \,\textrm{d}\mu ; \end{aligned} \end{aligned}$$

here, $\int R(u(x,y,z))\,\textrm{d}\mu$ is defined in (2); the function $v_{\varepsilon }(u)$ is a bell-shaped function defined as

$$\begin{aligned}&v_{\varepsilon }(u)\\&\quad =\left( \sqrt{(2 u-1)^2+\varepsilon }-1\right) H_{\varepsilon }\left( \sqrt{(2 u-1)^2+\varepsilon }-1\right) , \end{aligned}$$

where $H_{\varepsilon }(u)$ is the smoothed Heaviside function of the form

$$ H_{\varepsilon }(u)=\frac{1}{2}+\frac{1}{\pi }\arctan {\left( u/\varepsilon \right) }, $$

effectively constraining the range of *u* to lie in [0, 1];  the function ${\mathcal {D}}_G\left( x,\,y,\,z\right)$ is the geodesic distance function from the Roberts-Chen model, allowing users to specify foreground and background seeds, respectively, to guide the contour evolution. For example, if a single foreground point $(x_0,\,y_0,\,z_0)$ is specified, the geodesic distance function ${\mathcal {D}}$ satisfies:

$$\begin{aligned} |{\mathcal {D}}\left( x,\,y,\,z\right) |&=h(\nabla f(x,\,y,\,z)),\\ {\mathcal {D}}\left( x_0,\,y_0,\,z_0\right)&=0, \end{aligned}$$

where $h(\nabla f(x,\,y,\,z))$ is a function with respect to the image gradient. For example, if $h=1$, this reduces to the Euclidean distance.

To optimize the above energy, we focus on the regularization $\int R\left( u\left( x,\,y,\,z\right) \right) \,\textrm{d}\mu$ (see (1)). A direct approach to minimize (1) is to numerically solve the fourth-order partial differential equation derived from the Euler-Lagrange equation $\frac{\delta \int R(u)\,\textrm{d}\mu }{\delta u}.$ Such an approach has been widely adapted to energies involving 1D curvature, for instance, Euler’s Elastica. In practice, most studies (Zhu et al., 2013; Deng et al., 2019) routinely introduce appropriate auxiliary variables, splitting the problem into manageable subproblems. However, this numerical strategy typically places more emphasis on specific machinery and implementation details, which could eventually obscure the geometric nature of the problem and its solution at hand.

From the perspective of Riemannian geometry, it seems natural to replace the argument *u* of the energy by the metric tensor ${\textbf{g}}.$ Then, calculus of variations can be employed with respect to the $3\times 3$ metric tensor $\frac{\delta \int R({\textbf{g}})\,\textrm{d}\mu }{\delta {\textbf{g}}}$, which is known as the Einstein tensor (Einstein et al., 1916). However, recovering a parametric surface *u* from a metric tensor ${\textbf{g}}$ is generally an ill-posed problem due to the fact that different surfaces may share the same metric tensor. The Euclidean plane and cylinder, for example, share the same metric tensor (they are locally isometric) as cutting the cylinder along a generator and unrolling it onto the plane does not change pairwise geodesic distances between points.

Thus, it would be beneficial to explore other ways to handle this optimization. Note that due to the particular form of the parametric surface $(x,\,y,\,z,\,u(x,\,y,\,z))$, the metric tensor ${\textbf{g}}$ (3) is uniquely determined by the gradient $\nabla u$. Reconstructing images or surfaces from gradient fields is a well-studied problem in computer vision (see, e.g., Pérez et al. (2003)). However, recovering from ${\textbf{g}}$ poses a greater challenge as it might be impossible to solve for the gradient $\nabla u$ using the matrix ${\textbf{g}}$ even in the sense of least squares as in Borg and Groenen (2007). More precisely, given a known ${\textbf{g}}$, the system ${\textbf{I}}+\nabla u {\nabla u}^{\operatorname {T}}\approx {\textbf{g}}$ of six quadratic equations in the three unknown components of $\nabla u$ might not be solvable since, for example, the sign of $\nabla u$ cannot be determined, or the diagonal entries of ${\textbf{g}}$ might become smaller than 1. However, it is possible to avoid the challenging task of having to solve for the unknown gradient $\nabla u$ from a known metric tensor ${\textbf{g}}.$ We can directly derive the variational calculus with respect to the gradient $\nabla u$. That is, (9) can be reformulated as

10 $$\begin{aligned} \begin{aligned}&\min _{u,{\textbf{v}}}\int \lambda _1 {\left( f(x,\,y,\,z)-c_1\right) }^2u\left( x,\,y,\,z\right) \,\textrm{d}\Omega \\&\quad -\int \lambda _2{\left( f(x,y,z)-c_2\right) }^2u\left( x,\,y,\,z\right) \,\textrm{d}\Omega \\&\quad +\theta \int {\mathcal {D}}_G\left( x,\,y,\,z\right) u\left( x,\,y,\,z\right) \,\textrm{d}\Omega \\&\quad +\alpha \int \nu _\varepsilon \left( u\left( x,\,y,\,z\right) \right) \,\textrm{d}\Omega \\&\quad +\int {|\nabla u- {\textbf{v}}|}^2\,\textrm{d}\Omega + \int R\left( {\textbf{v}}\left( x,\,y,\,z\right) \right) \,\textrm{d}\mu , \end{aligned} \end{aligned}$$

following the idea that ${\textbf{v}}$ will resemble $\nabla u$ in the optimum. Note that, for the purpose of not overloading the notation, we do not introduce a new notation for *R* here. The notation $R\left( {\textbf{v}}\left( x,\,y,\,z\right) \right) ,$ i.e., for a vector valued function ${\textbf{v}},$ denotes the scalar curvature of the manifolds whose gradient field is ${\textbf{v}}.$

### Euler-Lagrange Equation

This section is dedicated to the Euler-Lagrange equation of (10) with respect to the two variables, *u* and its gradient ${\textbf{v}}$, whose components are denoted by $v_i$ in the following section.

The variational calculus with respect to *u* has been comprehensively described in Roberts et al. (2019), and we refer interested readers to the in-depth derivation. The resulting equation is the following:

11 $$\begin{aligned} \begin{aligned}&\bigtriangleup u - \nabla \cdot {\textbf{v}} -\theta {\mathcal {D}}_G\left( x,\,y,\,z\right) -\alpha \nu _\varepsilon '\left( u\right) \\&\quad -\lambda _1 \left( f\left( x,\,y,\,z\right) -c_1\right) +\lambda _2 \left( f\left( x,\,y,\,z\right) -c_2\right) =0. \end{aligned} \end{aligned}$$

As for ${\textbf{v}}$, the Euler-Lagrange equation is

12 $$\begin{aligned} 2\left( {\textbf{v}}-\nabla u\right) +\frac{\delta \int R\left( {\textbf{v}}\right) \textrm{d}\mu }{\delta {\textbf{v}}}=0, \end{aligned}$$

where $\frac{\delta \int R\left( {\textbf{v}}\right) \textrm{d}\mu }{\delta {\textbf{v}}}$ is the Euler-Lagrange equation of $\int R\left( {\textbf{v}}\right) \textrm{d}\mu$ with respect to ${\textbf{v}}$ to be discussed next.

We now focus on the variational calculus of ${\textbf{v}}$. To do this, we first express the matrix ${\textbf{g}}$ and the Hessian ${\textbf{H}}$ in (2) in terms of the gradient ${\textbf{v}}$ rather than *u*. It is straightforward to see that ${\textbf{g}}$ now takes the form

$$\begin{aligned} {\textbf{g}}={\textbf{I}}+{\textbf{v}}{\textbf{v}}^{\operatorname {T}}, \end{aligned}$$

and the Hessian matrix ${\textbf{H}}$ takes the form

$$\begin{aligned} {\textbf{H}}=\operatorname {D}{\textbf{v}}, \end{aligned}$$

where the notation $\operatorname {D}$ means generating the Jacobian matrix ${\textbf{H}}$ of the differentiable mapping ${\textbf{v}}$. We use the notation $v_{ij}$ to represent ${\textbf{H}}$’s components just for notational consistency with the previous notation $v_i$. That is, we use $v_i$ to represent the i-th element of ${\textbf{v}}$ and naturally use $v_{ij}$ to represent the partial derivative of $v_i$ relative to the j-th variable.

Observe that we do not develop a new formula for any of the above curvatures, but instead, we provide an interpretation in terms of the gradient ${\textbf{v}}$. From the gradient perspective, we see that the Einstein-Hilbert action (1) is now uniquely determined by the vector-valued function ${\textbf{v}}$. Based on this observation, it is sufficient to derive the Euler-Lagrange equation with respect to ${\textbf{v}}$, namely, investigating how $\int R({\textbf{v}})\textrm{d}\mu$ varies with ${\textbf{v}}$.

In calculus, this can be accomplished by finding a point ${\textbf{v}}$ at which all directional derivatives are zero, i.e., $\lim _{t\rightarrow 0}\frac{\textrm{E}({\textbf{v}}+t{\textbf{w}})-\textrm{E}({\textbf{v}})}{t}=0$ for all ${\textbf{w}}$, where $\textrm{E}$ represents our loss function (1) for convenience, ${\textbf{w}}$ is an arbitrary vector-valued function with the same size as ${\textbf{v}}$, and *t* is a small real value. This limit involves two steps: first, we move the point ${\textbf{v}}$ at the velocity $\frac{\partial {\textbf{v}}}{\partial t}={\textbf{w}}$ to reach a new point ${\textbf{v}}+t{\textbf{w}}$ after a short time *t*; then we calculate the deviation of energy from the initial energy. In variational calculus, we can consider the gradient field a function ${\textbf{v}}(t)$ of time *t*, and thus the energy can also be expressed as a function $\textrm{E}({\textbf{v}}(t))$ of *t*. At this point, variational calculus analyzes the derivative $\textrm{E}_t$ with ${\textbf{v}}$ having velocity ${\textbf{v}}_t={\textbf{w}}$. In the following section, for notational convenience, we omit the symbol ${\textbf{w}}$ and simply use ${\textbf{v}}_t$ to refer to it.

Assuming that ${\textbf{v}}$ varies with a time *t*, we can calculate $\frac{\partial \int R({\textbf{v}})\textrm{d}\mu }{\partial t}$ as

13 $$\begin{aligned} \begin{aligned}&\frac{\partial \int R\left( {\textbf{v}}\right) \textrm{d}\mu }{\partial t}\\&\quad =\frac{\partial \int \operatorname {tr}\left( {\textbf{g}}^{-1}\textbf{Ric}\right) \sqrt{\det \left( {\textbf{g}}\right) }\,\textrm{d}x\textrm{d}y\textrm{d}z}{\partial t}\\&\quad =\int R\frac{\partial \sqrt{\det \left( {\textbf{g}}\right) }}{\partial t}\,\textrm{d}x\textrm{d}y\textrm{d}z\\&\quad \quad +\int \operatorname {tr}\left( \textbf{Ric}\frac{\partial \left( {{\textbf{g}}}^{-1}\right) }{\partial t}\right) \sqrt{\det \left( {\textbf{g}}\right) }\,\textrm{d}x\textrm{d}y\textrm{d}z\\&\quad \quad +\int \operatorname {tr}\left( {{\textbf{g}}}^{-1}\frac{\partial \textbf{Ric}}{\partial t}\right) \sqrt{\det \left( {\textbf{g}}\right) }\,\textrm{d}x\textrm{d}y\textrm{d}z. \end{aligned} \end{aligned}$$

The first term of the right-hand side of (13) can be written as

$$\begin{aligned}&\int R\frac{\partial \sqrt{\det \left( {\textbf{g}}\right) }}{\partial t}\,\textrm{d}x\textrm{d}y\textrm{d}z\\&\quad =\int R\operatorname {tr}\left( {{\textbf{g}}}^{-1}{\textbf{v}}{\left( \frac{\partial {\textbf{v}}}{\partial t}\right) }^{\operatorname {T}} \right) \sqrt{\det \left( {\textbf{g}}\right) }\,\textrm{d}x\textrm{d}y\textrm{d}z, \end{aligned}$$

and the second term can be written as

$$\begin{aligned}&\int \operatorname {tr}\left( \textbf{Ric}\frac{\partial \left( {{\textbf{g}}}^{-1}\right) }{\partial t}\right) \sqrt{\det \left( {\textbf{g}}\right) }\,\textrm{d}x\textrm{d}y\textrm{d}z\\&\quad =\int \operatorname {tr}\left( -2{{\textbf{g}}}^{-1}{\textbf{v}}{\left( \frac{\partial {\textbf{v}}}{\partial t}\right) }^{\operatorname {T}}{{\textbf{g}}}^{-1}\textbf{Ric} \right) \\&\quad \sqrt{\det \left( {\textbf{g}}\right) }\,\textrm{d}x\textrm{d}y\textrm{d}z\\&\quad =\int \operatorname {tr}\left( -2{{\textbf{g}}}^{-1}\textbf{Ric}{{\textbf{g}}}^{-1}{\textbf{v}}{\left( \frac{\partial {\textbf{v}}}{\partial t}\right) }^{\operatorname {T}} \right) \\&\quad \sqrt{\det \left( {\textbf{g}}\right) }\,\textrm{d}x\textrm{d}y\textrm{d}z. \end{aligned}$$

By summing these two terms, we rewrite the right-hand side of (13), without its third term, as,

14 $$\begin{aligned} \begin{aligned}&\int R\frac{\partial \sqrt{\det \left( {\textbf{g}}\right) }}{\partial t}\,\textrm{d}x\textrm{d}y\textrm{d}z\\&\quad +\int \operatorname {tr}\left( \textbf{Ric}\frac{\partial \left( {{\textbf{g}}}^{-1}\right) }{\partial t}\right) \sqrt{\det \left( {\textbf{g}}\right) }\,\textrm{d}x\textrm{d}y\textrm{d}z\\&\quad =\int \operatorname {tr}\left( \left( R{\textbf{I}}-2{{\textbf{g}}}^{-1}\textbf{Ric}\right) {{\textbf{g}}}^{-1}{\textbf{v}}{\left( \frac{\partial {\textbf{v}}}{\partial t}\right) }^{\operatorname {T}} \right) \\&\quad \sqrt{\det \left( {\textbf{g}}\right) }\,\textrm{d}x\textrm{d}y\textrm{d}z\\&\quad =\int \operatorname {tr}\left( {{\textbf{g}}}^{-1}\left( R{\textbf{g}}-2\textbf{Ric}\right) {{\textbf{g}}}^{-1}{\textbf{v}}{\left( \frac{\partial {\textbf{v}}}{\partial t}\right) }^{\operatorname {T}} \right) \\&\quad \sqrt{\det \left( {\textbf{g}}\right) }\,\textrm{d}x\textrm{d}y\textrm{d}z. \end{aligned} \end{aligned}$$

Next, we will demonstrate that the third term on the right-hand side of (13) vanishes:

15 $$\begin{aligned} \int \operatorname {tr}\left( {{\textbf{g}}}^{-1}\frac{\partial \textbf{Ric}}{\partial t}\right) \sqrt{\det \left( {\textbf{g}}\right) }\,\textrm{d}x\textrm{d}y\textrm{d}z=0. \end{aligned}$$

We should mention that the following exposition contains essentially introductory material (e.g., Christoffel symbols and Levi-Civita connection), which will probably be known from Riemannian geometry. We include it here for completeness.

The above equation (15) is equivalent to

$$\begin{aligned} \int g^{ql}\frac{\partial R_{ql}}{\partial t} \sqrt{\det \left( {\textbf{g}}\right) }\,\textrm{d}x\textrm{d}y\textrm{d}z=0. \end{aligned}$$

To see this, we observe the following equation holds for the Ricci curvature tensor (6) and the proof can be found in Appendix C:

16 $$\begin{aligned} \begin{aligned}&R_{ql}=\frac{\partial g^{sk}v_sv_{ql}}{\partial k}+v_sv_{ql}g^{sa}g^{kb}v_{ak}v_b\\&\quad -\frac{\partial g^{sk}v_sv_{qk}}{\partial l}-v_sv_{qk}g^{sa}g^{kb}v_{al}v_b. \end{aligned} \end{aligned}$$

With this representation, we have

$$\begin{aligned} \begin{aligned}&g^{ql}\frac{\partial R_{ql}}{\partial t}=g^{ql}\frac{\partial ^2 g^{sk}v_sv_{ql}}{\partial k \partial t}+g^{ql} \frac{\partial v_sv_{ql}g^{sa}g^{kb}v_{ak}v_b}{\partial t}\\&\quad -g^{ql}\frac{\partial ^2g^{sk}v_sv_{qk}}{\partial l\partial t}-g^{ql}\frac{\partial v_sv_{qk}g^{sa}g^{kb}v_{al}v_b}{\partial t}. \end{aligned} \end{aligned}$$

Thus, recalling that $f\frac{\partial g}{\partial k}=\frac{\partial fg}{\partial k}-\frac{\partial f}{\partial k}g$, we obtain

17 $$\begin{aligned} \begin{aligned}&g^{ql}\frac{\partial R_{ql}}{\partial t}=\frac{\partial g^{ql} {\left( g^{sk}v_sv_{ql}\right) }_t}{\partial k}-\frac{\partial g^{sk}v_sv_{ql}}{\partial t}\frac{\partial g^{ql}}{\partial k}\\&\quad +g^{ql} \frac{\partial v_sv_{ql}g^{sa}}{\partial t}g^{kb}v_{ak}v_b+g^{ql}v_sv_{ql}g^{sa}\frac{\partial g^{kb}v_{ak}v_b}{\partial t} \\&\quad -\frac{\partial g^{ql}{\left( g^{sk}v_sv_{qk}\right) }_t}{\partial l}+\frac{\partial g^{sk}v_sv_{qk}}{\partial t}\frac{\partial g^{ql}}{\partial l}\\&\quad -g^{ql}\frac{\partial v_sv_{qk}g^{sa}}{\partial t}g^{kb}v_{al}v_b-g^{ql}v_sv_{qk}g^{sa}\frac{\partial g^{kb}v_{al}v_b}{\partial t}. \end{aligned} \end{aligned}$$

According to the Einstein summation convention, we can rename an index if it appears twice, such as $a_ib^i=a_jb^j$ or $a_{ij}b^{ij}=a_{ji}b^{ji}$. Thus, the seventh term of the right-hand side of (17) reads

$$\begin{aligned} -g^{ql}\frac{\partial v_sv_{qk}g^{sa}}{\partial t}g^{kb}v_{al}v_b=-\frac{\partial g^{sk}v_sv_{ql}}{\partial t}g^{qa}g^{lb}v_{ak}v_b, \end{aligned}$$

and the last term can be written as

$$\begin{aligned} -g^{ql}v_sv_{qk}g^{sa}\frac{\partial g^{kb}v_{al}v_b}{\partial t}=-\frac{\partial g^{sk}v_sv_{ql}}{\partial t}g^{qa}g^{lb}v_av_{bk}. \end{aligned}$$

The virtue of interchanging indices is that, at this point, three terms of (17) share a common factor, $\frac{\partial g^{sk}v_sv_{ql}}{\partial t}$. Additionally, by observing that the following equation holds

$$\begin{aligned} \frac{\partial g^{ql}}{\partial k}+g^{qa}g^{lb}v_{ak}v_b+g^{qa}g^{lb}v_av_{bk}=0, \end{aligned}$$

or in matrix form (Petersen & Pedersen, 2008)

$$\begin{aligned} \frac{\partial {\textbf{g}}^{-1}}{\partial k}=-{\textbf{g}}^{-1}\frac{\partial {\textbf{g}}}{\partial k} {\textbf {g}}^{-1}, \end{aligned}$$

we can conclude that the sum of the second, seventh, and eighth terms is zero. Analogously, the sum of the fourth and sixth terms is

$$\begin{aligned} -\frac{\partial g^{sk}v_sv_{qk}}{\partial t}g^{qa}g^{lb}v_{al}v_{b}. \end{aligned}$$

Therefore, the eight terms of (17) reduce to four terms:

$$\begin{aligned}&g^{ql}\frac{\partial R_{ql}}{\partial t}=\frac{\partial g^{ql} {\left( g^{sk}v_sv_{ql}\right) }_t}{\partial k}+\frac{\partial g^{sk}v_sv_{ql}}{\partial t}g^{ql}g^{ab}v_{ak}v_b\\&\quad -\frac{\partial g^{ql}{\left( g^{sk}v_sv_{qk}\right) }_t}{\partial l}-\frac{\partial g^{sk}v_sv_{qk}}{\partial t}g^{qa}g^{lb}v_{al}v_{b}. \end{aligned}$$

Since we aim to make the term $\int g^{ql}\frac{\partial R_{ql}}{\partial t} \sqrt{\det \left( {\textbf{g}}\right) }\,\textrm{d}x\textrm{d}y\textrm{d}z$ vanish, this can be achieved if $g^{ql}\frac{\partial R_{ql}}{\partial t} \sqrt{\det \left( {\textbf{g}}\right) }$ can emerge from a divergence. To see this, we first denote the vector $g^{ql}\frac{\partial g^{sk}v_sv_{ql}}{\partial t}$ by ${\textbf{a}}$ with components $a^k$ and $g^{ql}\frac{\partial g^{sk}v_sv_{qk}}{\partial t}$ by ${\textbf{b}}$ with components $b^l$. From the linear algebra identity

$$\begin{aligned} \frac{\partial \det \left( {\textbf{g}}\right) }{\partial k}=\det \left( {\textbf{g}}\right) g^{ab}\frac{\partial g_{ab}}{\partial k}=2\det \left( {\textbf{g}}\right) g^{ab}v_{ak}v_{b}, \end{aligned}$$

we have

$$\begin{aligned}&\phantom {=}g^{ql}\frac{\partial R_{ql}}{\partial t}\\&\quad =\frac{\partial a^k}{\partial k}+a^kg^{ab}v_{ak}v_b - \frac{\partial b^l}{\partial l}-b^ag^{lb}v_{al}v_{b}\\&\quad =\nabla \cdot {\textbf{a}}+\frac{{\textbf{a}} \cdot \nabla \det \left( {\textbf{g}}\right) }{2\det \left( {\textbf{g}}\right) } - \nabla \cdot {\textbf{b}}-\frac{{\textbf{b}} \cdot \nabla \det \left( {\textbf{g}}\right) }{2\det \left( {\textbf{g}}\right) }\\&\quad =\frac{1}{\sqrt{\det \left( {\textbf{g}}\right) }}\left( \sqrt{\det \left( {\textbf{g}}\right) } \nabla \cdot {\textbf{a}}+{\textbf{a}} \cdot \nabla \sqrt{\det \left( {\textbf{g}}\right) } \right) \\&\quad -\frac{1}{\sqrt{\det \left( {\textbf{g}}\right) }}\left( \sqrt{\det \left( {\textbf{g}}\right) } \nabla \cdot {\textbf{b}}+{\textbf{b}} \cdot \nabla \sqrt{\det \left( {\textbf{g}}\right) } \right) \\&\quad =\frac{\nabla \cdot \sqrt{\det \left( {\textbf{g}}\right) }{\textbf{a}}}{\sqrt{\det \left( {\textbf{g}}\right) }}-\frac{\nabla \cdot \sqrt{\det \left( {\textbf{g}}\right) }{\textbf{b}}}{\sqrt{\det \left( {\textbf{g}}\right) }}. \end{aligned}$$

Thus,

$$\begin{aligned}&\phantom {=}\int g^{ql}\frac{\partial R_{ql}}{\partial t}\sqrt{\det \left( {\textbf{g}}\right) }\,\textrm{d}x\textrm{d}y\textrm{d}z\\&\quad =\int \left( \frac{\nabla \cdot \sqrt{\det \left( {\textbf{g}}\right) }{\textbf{a}}}{\sqrt{\det \left( {\textbf{g}}\right) }}-\frac{\nabla \cdot \sqrt{\det \left( {\textbf{g}}\right) }{\textbf{b}}}{\sqrt{\det \left( {\textbf{g}}\right) }}\right) \\&\quad \sqrt{\det \left( {\textbf{g}}\right) }\,\textrm{d}x\textrm{d}y\textrm{d}z\\&\quad =\int \left( \nabla \cdot \sqrt{\det \left( {\textbf{g}}\right) }{\textbf{a}}-\nabla \cdot \sqrt{\det \left( {\textbf{g}}\right) }{\textbf{b}}\right) \,\textrm{d}x\textrm{d}y\textrm{d}z\\&\quad =0, \end{aligned}$$

where $\frac{\partial {\textbf{v}}}{\partial t}$ assumes a Dirichlet boundary condition, $\frac{\partial {\textbf{v}}}{\partial t}=0$, to make the above integral vanish.

Hence indeed the third term of the right-hand side of (13) vanishes, and from (14), we have

$$\begin{aligned} \begin{aligned}&\frac{\partial \int R\left( {\textbf{v}}\right) \textrm{d}\mu }{\partial t}=\int \sqrt{\det \left( {\textbf{g}}\right) }\\ &\quad \operatorname {tr}\left( {{\textbf{g}}}^{-1}\left( R{\textbf{g}}-2\textbf{Ric}\right) {{\textbf{g}}}^{-1}{\textbf{v}}{\left( \frac{\partial {\textbf{v}}}{\partial t}\right) }^{\operatorname {T}} \right) \,\textrm{d}x\textrm{d}y\textrm{d}z. \end{aligned} \end{aligned}$$

The stationary point ${\textbf{v}}$ of the energy satisfies $\frac{\partial \int R\left( {\textbf{v}}\right) \textrm{d}\mu }{\partial t}=0$, i.e.,

$$ \begin{aligned}&\int \operatorname {tr}\left( {{\textbf{g}}}^{-1}\left( R{\textbf{g}}-2\textbf{Ric}\right) {{\textbf{g}}}^{-1}{\textbf{v}}{\left( \frac{\partial {\textbf{v}}}{\partial t}\right) }^{\operatorname {T}} \right) \\&\quad \sqrt{\det \left( {\textbf{g}}\right) }\,\textrm{d}x\textrm{d}y\textrm{d}z=0. \end{aligned} $$

Observing that the metric tensor ${\textbf{g}}$ is a positive definite matrix assigned to each voxel, the following conditions hold: $\left( {\textbf{g}}\right) ^{-1}$ is a positive definite matrix, $\sqrt{\det \left( {\textbf{g}}\right) }> 0$, and for any vector ${\textbf{a}}$, ${{\textbf{a}}}^{\operatorname {T}}\left( {\textbf{g}}\right) ^{-1}{\textbf{a}}\ge 0$. Thus, if $\frac{\partial {\textbf{v}}}{\partial t}=\left( R{\textbf{g}}-2\textbf{Ric}\right) {{\textbf{g}}}^{-1}{\textbf{v}}$, the energy will increase.

This implies that the Euler-Lagrange equation $\frac{\delta \int R\left( {\textbf{v}}\right) \textrm{d}\mu }{\delta {\textbf{v}}}$ with respect to ${\textbf{v}}$ is

18 $$\begin{aligned} \left( R{\textbf{g}}-2\textbf{Ric}\right) {{\textbf{g}}}^{-1}{\textbf{v}}=0. \end{aligned}$$

For 3D manifold, $R{\textbf{g}}-2\textbf{Ric}$, namely the Einstein tensor (Einstein et al., 1916), being equal to 0 is locally equivalent to the 3D Euclidean space, the smoothest possible space.

At this point, the Euler-Lagrange equation (12) relative to ${\textbf{v}}$ becomes

19 $$\begin{aligned} 2\left( {\textbf{v}}-\nabla u\right) +\left( R{\textbf{g}}-2\textbf{Ric}\right) {{\textbf{g}}}^{-1}{\textbf{v}}=0. \end{aligned}$$

### Numerical Implementation

We have described the Euler-Lagrange equation of *u* in (11) and ${\textbf{v}}$ (i.e., $\nabla u$) in (19). In this section, we shall describe a numerical scheme for solving it.

We follow the additive operator splitting (AOS) strategy (see, e.g., Roberts et al. (2019)) for the subproblem (11) of *u*,

$$\begin{aligned} \frac{\partial u}{\partial t}=\Delta u-f, \end{aligned}$$

where, from (11), *f* reads

$$\begin{aligned}&\nabla \cdot {\textbf{v}} +\theta {\mathcal {D}}_G\left( x,\,y,\,z\right) +\alpha \nu '\left( u\right) \\&\quad +\lambda _1 {\left( f\left( x,\,y,\,z\right) -c_1\right) }^2 -\lambda _2 {\left( f\left( x,\,y,\,z\right) -c_2\right) }^2. \end{aligned}$$

The semi-implicit form is

$$ u^{k+1}={\left( {\textbf{I}}-\tau \Delta \right) }^{-1}\left( u^{k}-\tau f\right) . $$

Since the Laplacian is the sum of three convolutions (linear maps or matrices), namely, $\frac{\partial ^{2}}{\partial x^{2}}$, $\frac{\partial ^{2}}{\partial y^{2}}$, and $\frac{\partial ^{2}}{\partial z^{2}}$, by Taylor’s expansion $\frac{1}{1-x}=1+x+x^2+\cdots$, we obtain

$$\begin{aligned}&{\left( {\textbf{I}}-\tau \Delta \right) }^{-1}\\&\quad \approx {\textbf{I}}+\tau \frac{\partial ^{2}}{\partial x^{2}}+\tau \frac{\partial ^{2}}{\partial y^{2}}+\tau \frac{\partial ^{2}}{\partial z^{2}}\\&\quad = \frac{{\textbf{I}}+3\tau \frac{\partial ^{2}}{\partial x^{2}}+{\textbf{I}}+3\tau \frac{\partial ^{2}}{\partial y^{2}}+{\textbf{I}}+3\tau \frac{\partial ^{2}}{\partial z^{2}}}{3}\\&\quad \approx \frac{{({\textbf{I}}-3\tau \frac{\partial ^{2}}{\partial x^{2}} )}^{-1}+{({\textbf{I}}-3\tau \frac{\partial ^{2}}{\partial y^{2}} )}^{-1}+{({\textbf{I}}-3\tau \frac{\partial ^{2}}{\partial z^{2}}) }^{-1}}{3}. \end{aligned}$$

The original 3D equation is thus decomposed into three separate 1D equations:

20 $$\begin{aligned} u^{k+1}=\frac{1}{3}\sum _{l=0}^{2}{\left( {\textbf{I}}-3\tau \frac{\partial ^{2}}{\partial l^{2}}\right) }^{-1}\left( u^{k}-\tau f\right) , \end{aligned}$$

where each 1D equation becomes a tridiagonal system of equations which can be solved with linear computational complexity.

An important force guiding *u* is ${\mathcal {D}}_G\left( x,\,y,\,z\right)$, composed of two distinct distances. They are denoted by ${\mathcal {D}}_{M}(x,\,y,\,z)$ and ${\mathcal {D}}_{AM}(x,\,y,\,z)$, representing the distances from the current voxel $(x,\,y,\,z)$ to the foreground and background seeds, respectively. We employ Dijkstra’s algorithm (Dijkstra, 1959) to calculate them, set ${\mathcal {D}}_G\left( x,\,y,\,z\right) =\frac{{\mathcal {D}}_{AM}}{{\mathcal {D}}_M+{\mathcal {D}}_{AM}}$ and scale it to the range $[-1,\,1]$ such that it can drive the contour according to its sign. Since the Matlab framework of the Spencer-Chen model is available on GitHub, we adapt the original Matlab code to update *u*.

For the ${\textbf{v}}$-subproblem, we update ${\textbf{v}}$ according to (19) as

$$ \begin{aligned} \frac{\partial {\textbf{v}}}{\partial t}&= 2\left( {\textbf{v}}-\nabla u\right) +\left( R{\textbf{g}}-2\textbf{Ric}\right) {{\textbf{g}}}^{-1}{\textbf{v}}. \end{aligned} $$

Its semi-implicit form involves solving a general linear system for a large 3D function, which is computationally prohibitive. In practice, we interpret the corresponding energy $\int {|\nabla u- {\textbf{v}}|}^2\,\textrm{d}\Omega + R\left( {\textbf{v}}\left( x,\,y,\,z\right) \right) \,\textrm{d}\mu$ in (10) as a homogeneous system of differential equations,

$$ {\left\{ \begin{array}{ll} \frac{\partial {\textbf{v}}}{\partial t}& =\left( R{\textbf{g}}-2\textbf{Ric}\right) {{\textbf{g}}}^{-1}{\textbf{v}}\\ {{\textbf{v}}}(0)& =\nabla u, \end{array}\right. } $$

whose solution is given by

$$\begin{aligned} \begin{aligned} {\textbf{v}}\left( t\right) =e^{\left( R{\textbf{g}}-2\textbf{Ric}\right) {{\textbf{g}}}^{-1}t} \nabla u, \end{aligned} \end{aligned}$$

yielding the following update strategy for ${\textbf{v}}$

21 $$\begin{aligned} {\textbf{v}}^{k+1}=e^{\left( R{\textbf{g}}-2\textbf{Ric}\right) {{\textbf{g}}}^{-1}t} \nabla u^{k}, \end{aligned}$$

where $t>0$ is a parameter, and the metric tensor ${\textbf{g}}$ (3), Ricci curvature tensor $\textbf{Ric}$ (6) and the scalar curvature *R* (8) can be computed using $u^{k}$. Here, *t* controls the distance between the new gradient ${\textbf{v}}$ and the preceding gradient $\nabla u,$

We employ the finite difference method to calculate all derivatives, PyTorch’s einsum function to perform tensor operations, and PyTorch’s matrix_exp function to compute the matrix exponential (21).

## Existence and Uniqueness of the Viscosity Solution

Based on Crandall et al. (1992); Ishii and Sato (2004), we can investigate the solution of the partial differential equation $F(x,u,Du,D^2u)=0$, where *x* denotes the point in ${\mathbb {R}}^n$, $u \in {\mathbb {R}}$ represents the (relaxed) level set function, $Du\in {\mathbb {R}}^n$ is the gradient of *u*, $D^2u$ represents the $n\times n$ Hessian matrix, and *F* is a map from these values to a scalar. In addition, we denote $n\times n$ symmetrical matrices by ${\mathscr {M}}^{n}$. We follow the same notation convention as in Roberts et al. (2019) to describe the following theorem.

### [Style2 Style3 Style3]Theorem 1

(Theorem 2 in Roberts et al. (2019)) Assuming that conditions (C1)-(C2) and (I1)-(I7) are satisfied, then for each $u_0 \in$ $C({\bar{\Omega }})$, there exists a unique viscosity solution $u \in C([0, T) \times {\bar{\Omega }})$ of (22) and (23) that satisfies (24).

22 $$\begin{aligned}&\frac{\partial u}{\partial t}+F\left( t, x, u, D u, D^2 u\right) =0 \text { in } Q=(0, T) \times \Omega \end{aligned}$$

23 $$\begin{aligned}&B(x, D u)=0 \text { in } S=(0, T) \times \partial \Omega \end{aligned}$$

24 $$\begin{aligned}&u(0, x)=u_0(x) \text { for } x \in {\bar{\Omega }} \end{aligned}$$

**Conditions (C1)-(C2)**

1. $$F(t, x, u, p, X) \le F(t, x, v, p, X)$$ for $$u \le v$$.
2. $$F(t, x, u, p, X) \le F(t, x, u, p, Y)$$ for $$X, Y \in {\mathscr {M}}^n$$ and $$Y \le X$$.

**Conditions (I1)-(I7)**

Assume $\Omega$ is a bounded domain in ${\mathbb {R}}^n$ with $C^1$ boundary.

1. $$F \in C\left( [0, T] \times {\bar{\Omega }} \times {\mathbb {R}} \times \left( {\mathbb {R}}^n \backslash \{0\}\right) \times {\mathscr {M}}^n\right) $$.
2. There exists a constant $\gamma \in {\mathbb {R}}$ such that for each $(t, x, p, X) \in [0, T] \times {\bar{\Omega }} \times \left( {\mathbb {R}}^n \backslash \{0\}\right) \times {\mathscr {M}}^n$, the function $u \mapsto F(t, x, u, p, X)-\gamma u$ is non-decreasing on ${\mathbb {R}}$.
3. *F* is continuous at (*t*, *x*, *u*, 0, 0) for any $(t, x, u) \in$ $[0, T] \times {\bar{\Omega }} \times {\mathbb {R}}$ in the sense that
  $$ -\infty<F_*(t, x, u, 0,0)=F^*(t, x, u, 0,0)<\infty $$
  holds. Here, $F^*$ and $F_*$ denote, respectively, the upper and lower semi-continuous envelopes of *F*, which are defined on $[0, T] \times {\bar{\Omega }} \times {\mathbb {R}} \times {\mathbb {R}}^n \times {\mathscr {M}}^n$.
4. $B \in C\left( {\mathbb {R}}^n \times {\mathbb {R}}^n\right) \cap C^{1,1}\left( {\mathbb {R}}^n \times \left( {\mathbb {R}}^n \backslash \{0\}\right) \right)$, where $C^{1,1}$ is the Hölder functional space.
5. For each $x \in {\mathbb {R}}^n$, the function $p \mapsto B(x, p)$ is positively homogeneous of degree one in *p*, i.e. $B(x, \lambda p)=$ $\lambda B(x, p)$ for all $\lambda \ge 0$ and $p \in {\mathbb {R}}^n \backslash \{0\}$.
6. There exists a positive constant $\Theta$ such that $\langle {\varvec{n}}(x), D_p B(x, p)\rangle \ge \Theta$ for all $x \in \partial \Omega$ and $p \in {\mathbb {R}}^n \backslash \{0\}$. Here, ${\varvec{n}}(x)$ denotes the unit outward normal vector of $\Omega$ at $x \in \partial \Omega$.
7. For each $R>0$, there exists a non-decreasing continuous function $\omega _R:[0, \infty ) \rightarrow [0, \infty )$ satisfying $\omega _R(0)=0$ such that if $X, Y \in {\mathscr {M}}^n$ and $\mu _1, \mu _2 \in$ $[0, \infty )$ satisfy
  25 $$\begin{aligned} \left[ \begin{array}{ll} X & 0 \\ 0 & Y \end{array}\right] \le \mu _1\left[ \begin{array}{cc} I & -I \\ -I & I \end{array}\right] +\mu _2\left[ \begin{array}{ll} I & 0 \\ 0 & I \end{array}\right] \end{aligned}$$

then

$$ \begin{aligned}&F(t, x, u, p, X)-F(t, y, u, q,-Y) \ge \\&\quad -\omega _R\left( \mu _1\left( |x-y|^2+\rho (p, q)^2\right) +\mu _2+|p-q|\right. \\&\quad +|x-y|(\max (|p|,|q|)+1)) \end{aligned} $$

for all $t \in [0, T], x, y \in {\bar{\Omega }}, u \in {\mathbb {R}}$, with $|u| \le R$, $p, q \in {\mathbb {R}}^n \backslash \{0\}$ and $\rho (p, q)=\min \left( \frac{|p-q|}{\min (|p|,|q|)}, 1\right)$.

We now turn to the uniqueness of the solution of (11). Prior to applying Theorem 1, we first rewrite (11) as

26 $$\begin{aligned} \begin{aligned} F(x, u, p, X)=-\operatorname {tr}\left( X\right) +k(u)+f(x)\\ \end{aligned} \end{aligned}$$

where $$k(u)=\alpha v_{\varepsilon }^{\prime }(u)$$ and $$f(x)=\lambda _1 {\left( f\left( x\right) -c_1\right) }^2 -\lambda _2 {\left( f\left( x\right) -c_2\right) }^2+\theta {\mathcal {D}}_G\left( x\right) +\nabla \cdot {\textbf{v}}\left( x\right) $$.

### [Style2 Style3 Style3]Theorem 2

The parabolic PDE $\frac{\partial u}{\partial t}+F\left( t, x, u, D u, D^2 u\right) =0$ with $u_0=u(0, x) \in$ $C({\bar{\Omega }}), F$ as defined in (26) and Neumann boundary conditions has a unique solution $u=u(t, x)$ in $C([0, T) \times {\bar{\Omega }})$.

### Proof

To apply Theorem 1, we need to show that *F* satisfies (C1)-(C2) and (I1)-(I7).

1. To satisfy (C1), we need to show that $k\left( u\right)$ is non-decreasing, which can be easily verified through a plot of function *k* of *u*.
2. If $Y\le X$, $\operatorname {tr}\left( Y\right) -\operatorname {tr}\left( X\right) =\operatorname {tr}\left( Y-X\right) \le 0$, which satisfies C2.
  1. *F* does not have singularities, and thus it is continuous and satisfies this condition.
  2. In *F*, the only term which depends on *u* is $k(u)=$ $\alpha v_{\varepsilon }^{\prime }(u)$. This condition requires $\alpha v'_\epsilon -\gamma u$ to be non-decreasing. Since in (C1) it has been shown that *k*(*u*) is non-decreasing, we can choose an arbitrary $\gamma <0$ to satisfy this condition.
  3. *F* is continuous so the upper and lower semi-continuous envelopes are equal. Because a continuous function from a compact space into a metric space is bounded, $f\left( x\right)$ defined on the 3D image is bounded, and $k\left( u\right)$ defined on the compact interval $[0,\,1]$ is bounded. Beyond $[0,\,1]$, observing $\lim \limits _{u\rightarrow +\infty }k(u)=2$ and $\lim \limits _{u\rightarrow -\infty }k(u)=-2$, $k\left( u\right)$ is also bounded. So its upper and lower semi-continuous envelopes are bounded.
  4. The Euler-Lagrange equations give Neumann boundary conditions
    $$ B(x, \nabla u)=\langle {\varvec{n}}, \nabla u-{\textbf{v}}\rangle =0 $$
    on $\partial \Omega$, where ${\varvec{n}}$ is the outward unit normal vector. In each iteration, we can set the Neumann boundary condition for $\nabla u$ and thus ${\textbf{v}}$ in fact also satisfies the Neumann boundary condition $\langle {\textbf{n}},\,{\textbf{v}}\rangle$ because we assume $\frac{\partial {\textbf{v}}}{\partial t}=0$ on $\partial \Omega$. At this time, the above equations become
    $$ B(x, \nabla u)=\langle {\varvec{n}}, \nabla u\rangle =0. $$
    Since the function *B* is a differentiable function, and its derivatives are defined on a compact set, $\nabla u$ is also bounded. Thus, we see that $B(x, \nabla u) \in C^{1,1}\left( {\mathbb {R}}^n \times {\mathbb {R}}^n \backslash \{0\}\right)$ and therefore this condition is satisfied.
  5. By the definition above, $B(x, \lambda \nabla u)=\langle {\varvec{n}}, \lambda \nabla u\rangle =$ $\lambda \langle {\varvec{n}}, \nabla u\rangle =\lambda B(x, \nabla u)$. So this condition is satisfied.
  6. As before, we can use the definition, $\langle {\varvec{n}}(x)$, $D_p \left. B(x, p)\right\rangle =\langle {\varvec{n}}(x), {\varvec{n}}(x)\rangle =|{\varvec{n}}(x)|^2$. So we can choose $\Theta =1$ and the condition is satisfied.
  7. (25) implies, for each vector *r* and *s*, we have
    $$\begin{aligned}&r^T X r+s^T Y s \\&\quad \le \mu _1\left[ \begin{array}{ll}r^T&s^T\end{array}\right] \left[ \begin{array}{cc}I & -I \\ -I & I\end{array}\right] \left[ \begin{array}{l}r \\ s\end{array}\right] \\ & +\mu _2\left[ \begin{array}{ll}r^T&s^T\end{array}\right] \left[ \begin{array}{ll}I & 0 \\ 0 & I\end{array}\right] \left[ \begin{array}{l}r \\ s\end{array}\right] \\&\quad = \mu _1|r-s|^2+\mu _2\left( |r|^2+|s|^2\right) .\end{aligned}$$
    and the trace can be represented as
    $$\begin{aligned} \operatorname {tr}\left( X\right) +\operatorname {tr}\left( Y\right) =\sum _{i=0}^2 \left( e_i^TXe_i+e_i^TYe_i\right) \le 6\mu _2. \end{aligned} $$
    Therefore,
    $$ \begin{aligned}&-(F(t,x, u, p,X)- F(t, y,u,q,-Y))\\&\quad =\operatorname {tr}\left( X\right) +\operatorname {tr}\left( Y\right) -f(x)+f(y)\\&\quad \le 6\mu _2+C|x-y| \end{aligned} $$
    where $f'(x)$ on the compact set $\Omega$ is bounded by a constant *C*, $|f'(x)|<C$. To satisfy (I7), we can set $\omega _R=\max \left( 6,\frac{C}{\max \left( |p|,|q|\right) +1}\right)$.$\square$

## Experimental Results

In this section, we compare our model with relevant models on three public datasets, then we demonstrate an ablation study to discuss how to select the best *t* parameter in (21).

The models under comparison are:

- **RC**: the 3D version of the Roberts-Chen Model (Section 2.3),
- **SUNETR**: Swin UNETR (Hatamizadeh et al., 2021),
- **SAM**: Segment Anything (Kirillov et al., 2023),
- **ZC**: 3D Zhang-Chen model (Zhang et al., 2015),
- **GrabCut**: 3D GrabCut model (Rother et al., 2004),
- **Ricci**: our proposed model based on the Ricci curvature tensor.

The SUNETR is a 3D transformer-based network for 3D medical image segmentation, which employs locally shifted windows (Swin) to mitigate the computational complexity introduced by the preceding global transformer. It ranks among the top-performing models for various datasets. SAM, recognized as a fundamental segmentation network by Meta AI, can segment "anything", requiring a few foreground and background seeds but no additional training data. The ZC model is a 3D variational model that utilizes markers near the object boundary to guide contour evolution. The GrabCut model is a classical graph-based approach. In principle, it can segment images of any dimension, as the number of dimensions only affects the number of edges adjacent to graph nodes. Our model, Ricci, is based on the RC model, replacing the traditional gradient-based regularization with the proposed Ricci curvature tensor-based regularization. We assigned the same seeds to both models to facilitate a fair comparison.

We employed three metrics: intersection over union (IoU) (Jaccard, 1912), Hausdorff distance (HD) (Rockafellar & Wets, 2009), and Dice score (DSC) (Dice, 1945); recall that the latter compares two sets *X* and *Y* of voxels by setting $\text {DSC}(X,Y)=2|X\cap Y|/(|X|+|Y|).$ As implementations we used MONAI’s MeanIoU, HausdorffDistanceMetric, and DiceMetric.

We evaluated our model on the following three tasks and data sets: the brain tumour core segmentation on the Brain Tumor Segmentation (BraTS) Challenge 2021 dataset (Bakas et al., 2018), the oesophagus and stomach segmentation on the Beyond the Cranial Vault (BTCV) Segmentation Challenge dataset (Landman et al., 2015), and the post-resection cavity segmentation on the REtroSpective Evaluation of Cerebral Tumors (RESECT) dataset (Xiao et al., 2017). These data sets consist of MRI, CT, and Ultrasound images (US), respectively. Each dataset is described in detail in its corresponding section below.

### BraTS Dataset

This section evaluates the brain tumour core segmentation on the BraTS dataset. We randomly selected 50 cases for evaluation from a total number of 1252 cases of this dataset. We first describe our implementation settings, then visualize the segmentation results on two representative cases, and finally summarise the results using the three metrics.

The Brain Tumor Segmentation (BraTS) Challenge 2021 dataset (Bakas et al., 2018), a collection of multi-institutional pre-operative baseline multi-parametric magnetic resonance imaging (mpMRI) scans, is set up to test state-of-the-art methods for the segmentation of heterogeneous brain glioblastoma sub-regions in mpMRI scans. Each batch of the dataset is a 4-channel 3D image: the t1-weighted scans (t1) are particularly useful for visualizing areas where the blood-brain barrier is intact; the t1-weighted scans with contrast enhancement (t1c) help highlight areas where the blood-brain barrier is disrupted; the t2-weighted scans (t2) are sensitive to fluid content and can help to visualize edema (swelling); the fluid attenuated inversion recovery scans (flair) are a type of T2 scan that suppresses the signal from free fluid, particularly useful for visualizing lesions surrounded by cerebrospinal fluid. Each annotation of the dataset comprises four different labels: background (label 0), necrotic and non-enhancing tumour core (label 1), peritumoral oedema (label 2), and enhancing tumour (label 4). Thus, the tumour core that we segment corresponds to label 1.

The PyTorch implementation of the SUNETR model and its weights on the BraTS dataset are available online on the MONAI Research Contributions (Cardoso et al., 2022). According to the original paper, the authors evaluated SUNETR’s performance through five-fold cross-validation. Here, we used the network weights corresponding to the first fold and compared all models on 50 cases of this fold.

The ZC model employs markers near the object to penalize contours that are far from object boundaries. The Matlab code for this model is available online. We first calculated a signed distance function to the ground truth boundary using Matlab’s bwdist and randomly sampled markers within a distance of less than 5. The *x*, *y*, and *z* coordinates of the selected prompts were then fed into the algorithm. We noted that the original code only worked with single-channel 3D images. Therefore, we adapted the region term to the $\ell ^2$ norm of vectors of length 4, representing four-channel images.

The GraphCut model has several variants in different packages. We used Matlab’s grabcut function, which took four inputs: the original image, the 3D superpixel, and the foreground and background markers. Since the original image was assumed to be a single-channel grayscale image, we only employed the first channel of the image. Throughout the experiments, we set the superpixel number to a fixed value of 100, 000. We used the same foreground and background markers as the RC and Ricci models.

SAM does not require a training set to train a model but restricts the input image to a 3-channel 8-bit 2D image. Among the four modalities of the BraTS dataset, we found the T1c display visible contrast, so we chose it as input. From the other three modalities, we chose flair and t1. We then cropped the 3-channel image to size $3\times 128\times 128\times 128$ and normalized intensities to lie in the interval $[0,\,255]$. We looped through each slice and randomly sampled three foreground seeds in label 1 and 20 background seeds in label 4 per loop iteration. However, the background seeds appear to encourage, rather than force, the model to recognize the background leading to an overestimation of the foreground and hence to a high recall with low precision. To deal with this, we used simple yet powerful post-processing for this and the following dataset. We performed a morphological opening on the binary result using a sphere kernel with radius 2 and then extracted its largest connected component.

We now turn to the proposed Ricci model and the RC model. For each of the four channels, we calculated the regional statistical information, ${\left( f(x,\,y,\,z)-c_1\right) }^2$ and ${\left( f(x,y,z)-c_2\right) }^2$, separately and then fed their mean into the region term. The corresponding coefficients were set to $\lambda _1=0.1$ and $\lambda _2=0.1$, since, in general, the foreground and background were not homogeneous. When executing Dijkstra’s algorithm to calculate the geodesic distance to two groups of seeds, the edge weight was set to equal the sum of absolute differences of adjacent voxels over the four channels. The Ricci model exhibits a parameter *t* in (21), which can be interpreted as controlling the step length of the gradient descent. When $t=0$, the updated gradient remains unchanged; the corresponding manifold will move toward a flat manifold as *t* increases. On the other hand, due to the existence of the exponential function in (21), the time increment should be limited to a specific range. In practice, we found the interval $[0,\,20]$ applicable. The remaining parameter is the iteration number of the model, which approximately depends on the foreground size. For this dataset, we assigned a constant value of 15 to the iteration number.

**Fig. 2:**
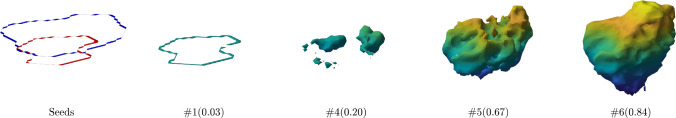
Intermediate contour evolution of the Ricci model for the necrotic tumour core segmentation on a representative image of the BraTS dataset. The foreground and background seeds (left figure) are shown in red and blue, respectively. They are located in two separate slices of the 3D image. The subcaptions of the form #$\alpha$($\beta$) indicate the iteration number ($\alpha$) and the corresponding DSC ($\beta$). The final result is shown in Figure 3

**Fig. 3:**
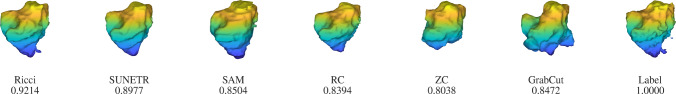
Segmentation results of all models and the ground truth for the case from Figure 2 (numbers representing the respective DSC). All models show good performances as there is high contrast between the necrotic tumour core (label 1) and GD-enhancing tumour (label 4) in the image

**Fig. 4:**
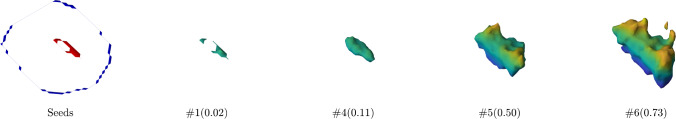
Intermediate contour evolution of the Ricci model for the necrotic tumour core segmentation on a more challenging image of the BraTS dataset. The foreground and background seeds reside in the same slice of the 3D image. The final result is shown in Figure 5

**Fig. 5:**
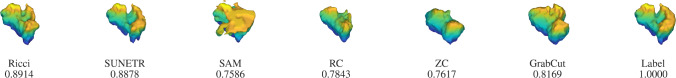
Segmentation results of all models and the ground truth for the case from Figure 4

Figure 2 demonstrates the seed configuration and intermediate contour evolution of the Ricci model for a representative 3D brain image. In the first figure (from the left), the red curve represents the foreground seeds, and the blue curve represents the background seeds. These foreground seeds are located in label 1 of a particular slice, and the background seeds lie in label 4 of the same slice. From the second to the last figure, the symbol, such as $\#1(0.03)$, denotes that the Ricci model reaches a DSC of 0.03 in the first iteration. It can be seen that the initial contour starts from the foreground seeds and then gradually grows outward. The contour expands rapidly initially, primarily due to the prior information that users fed into the geodesic distance term ${\mathcal {D}}_G$ through the foreground seeds and background seeds. In this case, the region with ${\mathcal {D}}_G>0$ has a DSC of 0.7063, which is surpassed in the sixth iteration (DSC: 0.84), mainly attributed to the regularization.

Figure 3 shows the segmentation results of all models and the ground truth for the above case from Figure 2. The proposed Ricci model reaches the highest DSC of 0.9214, demonstrating its effectiveness. Although SAM is a 2D model, it shows comparable performance (DSC: 0.8504) to the second-best, SUNETR (DSC: 0.8977). Notably, although the RC model was originally intended for 2D segmentation, it also gives a respectable result (DSC: 0.8394).

Figure 4 shows the foreground seeds, background seeds, initial contour, contour evolution, and corresponding DSC for another challenging case. Analogous to the preceding case, we define a curve in label 1 and, respectively, label 4 as the foreground and background seeds. The initial contour resembles the foreground seeds and gradually expands until it achieves a satisfactory result.

Figure 5 shows the segmentation results of all models and the ground truth for the same case as in Figure 4. It can be seen that SAM produces some broken and spurious parts, mainly due to the fact that SAM is a 2D model not utilizing information across different slices. Since the contrast around the boundary of Label 1 and 4 is lower compared to the preceding case (Figure 3), the gradient-based regularization in the RC model can not perform as well as before, leading to a DSC of 0.7843.

Table 1 summarizes the performance of the models employing the metrics: IoU, HD, and DSC. The Ricci model achieves the highest scores in IoU and DSC, with values of 0.8176 and 0.8989, respectively. Our model demonstrates a significant DSC improvement of 0.0304 over the SUNETR model and 0.0791 over the third-ranked 3D GrabCut, underscoring its efficiency. Compared to the RC model, the Ricci model demonstrates improvements of 0.1715, 0.7734, and 0.1145 across the three metrics, respectively. Although the RC model reaches a DSC (0.7844) similar to SAM (0.7833), the RC model’s HD is low. This may be attributed to its regularization, which is designed to eliminate noise from the segmentation. However, the RC model’s gradient-based regularization can not reliably recognize low contrast boundaries between Labels 1 and 4, and is therefore outperformed by the Ricci curvature-based regularization.

### BTCV Dataset

This section validates the proposed Ricci model for the oesophagus and stomach segmentation on six cases of the Beyond the Cranial Vault (BTCV) Segmentation Challenge dataset (Landman et al., 2015). For each organ, we visualize one representative segmentation result and give a quantitative summary in Table 1.

**Table 1:** Comparison of different models across four organs using three metrics, intersection over union (IoU), Hausdorff distance (HD), and Dice score (DSC)

	IoU	HD	DSC
	Brain Tumor Core		
Ricci	$\mathbf {0.8176}$	5.7077	$\mathbf {0.8989 }$
SUNETR	0.7686	$\mathbf {5.4127}$	0.8685
SAM	0.6524	13.6864	0.7833
RC	0.6461	6.4811	0.7844
ZC	0.6239	9.6484	0.7639
GrabCut	0.7004	7.1424	0.8198
	Esophagus		
Ricci	$\mathbf {0.6813}$	$\mathbf { 4.9998 }$	$\mathbf { 0.8097 }$
SUNETR	0.5710	20.7295	0.7245
SAM	0.4224	13.3435	0.5878
RC	0.5775	7.8754	0.7315
ZC	0.5786	6.0854	0.7293
GrabCut	0.3400	13.3435	0.5030
	Stomach		
Ricci	$\mathbf {0.8059}$	$\mathbf {7.8748}$	$\mathbf { 0.8918 }$
SUNETR	0.6366	67.4389	0.7562
SAM	0.7882	16.4916	0.8726
RC	0.6891	12.6888	0.8153
ZC	0.7002	17.4619	0.8211
GrabCut	0.7017	17.8386	0.8185
	Post-resection Cavity		
Ricci	$\mathbf {0.8304}$	$\mathbf {15.5260}$	$\mathbf { 0.9067 }$
SUNETR	0.7947	34.4419	0.8848
SAM	0.5769	23.0178	0.7220
RC	0.6909	24.5955	0.8164
ZC	0.6736	16.0370	0.8016
GrabCut	0.6077	48.0842	0.7519

The BTCV Segmentation Challenge focuses on segmenting abdomen organs across 50 abdomen CT images, each containing 13 organs. Of the 50 CT scans, 30 form the training sets with annotation, and the remaining scans form a test set without annotation. The CT scans have a fixed width and height of $512\times 512$, but the number of slices varies between 85 and 198.

The BTCV dataset is much smaller than the BraTS dataset (1252 scans). Therefore, SUNETR was initialized with self-supervised weights and then fine-tuned on the BTCV dataset (Tang et al., 2022; Cardoso et al., 2022). The PyTorch implementation of the network and the self-supervised weights were downloaded from the MONAI Research Contributions platform (Project-MONAI), and the final trained weights were downloaded from the MONAI Model Zoo (Cardoso et al., 2022). Since we trained on 24 scans, we used the remaining six validation scans to compare our model with the other models.

For SAM, we first cropped the 3D image to size $96\times 96 \times 96$, then normalized the input data to the grayscale range [0, 255], and repeated this for the other two channels to form a tensor of size $3\times 96\times 96 \times 96$. Since the oesophagus is larger than the above brain tumour, we randomly sampled, for each slice, the foreground to form 20 foreground seeds and randomly selected 20 background seeds from the background. It should be noted that the seed setting of the SAM model differs from our model because the former is a 2D model requiring a slice-by-slice seed selection.

The ZC, GrabCut, and our models used the same configuration as for the BraTS dataset, with some exceptions. Since BTCV has one channel, we used the original ZC code designed for single-channel 3D images. The number of Ricci’s iterations was set to 25 for this dataset due to its relatively large volume compared to brain tumours.

**Fig. 6:**
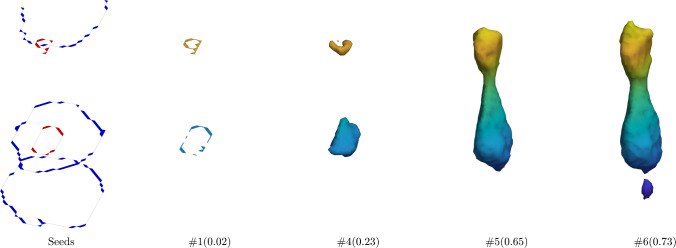
Intermediate contour evolution of the Ricci model for the oesophagus segmentation on a representative image of the BTCV dataset. The final result is shown in Figure 7

Figure 6 shows the foreground seeds, background seeds, initial contour, contour evolution, and corresponding DSC for a representative case of the oesophagus segmentation. It can be seen that the seeds are composed of two red foreground curves and three blue background curves. In the sixth iteration, the Ricci model reaches a DSC of 0.73.

**Fig. 7:**
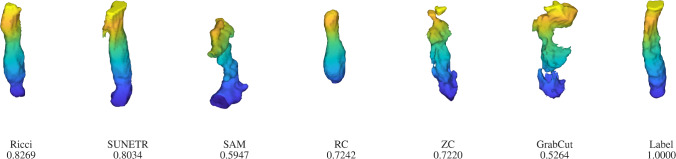
Segmentation results of all models and the ground truth for the case from Figure 6. SUNETR finds an additional component, which is not depicted here as it is far beyond the viewport. The SAM result was post-processed by a morphological opening followed by an extracting of the largest connected component resulting in a smoothed segmentation with improved DSC

Figure 7 visualizes all segmentation results for the case shown in Figure 6. The SUNETR model introduces a protruding branch at the top of the mesh. The SAM and GrabCut models produce two artificial branches at the top and bottom. RC and ZC models share a similar DSC of around 0.72, with RC displaying a smoother appearance. This is because the region of ${\mathcal {D}}_G>0$ has a DSC of only 0.1812, covering many irrelevant organs and tissues. Therefore, we increase the regularization effect by using a large $\gamma$ to excessively smooth the segmentation result, which reduces its DSC. In contrast, our proposed Ricci model achieves the highest DSC of 0.8269, with an improvement of 0.0235 over the second-best SUNETR.

**Fig. 8:**
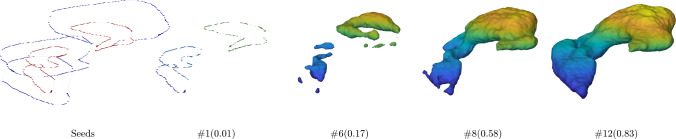
Intermediate contour evolution of the Ricci model on the stomach segmentation of the BTCV dataset. The final result is shown in Figure 9

Figure 8 illustrates the seed points of the Ricci model and the corresponding contour evolution for the stomach segmentation. As the stomach volume is much larger than the previous two organs, the resulting evolution speed is slightly slower than for the previous two organs reaching a DSC of 0.83 at the 12th iteration.

**Fig. 9:**
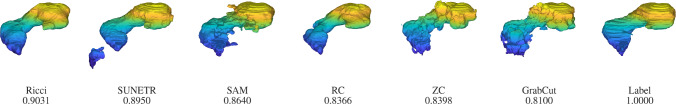
Segmentation results of all models and the ground truth for the case from Figure 8. (The SAM result was post-processed as before.)

Figure 9 visualizes the results of all models for the above case from Figure 8. Deep learning methods aim at optimizing a loss function (e.g., Dice loss) to impose geometric constraints. However, these constraints may not always be enforced, as highlighted by the presence of some unnecessary parts introduced by SUNETR (DSC: 0.8950) and SAM models (DSC: 0.8640). Traditional models aim to improve metrics through extensive geometric regularization, which is also challenging to achieve, as confirmed by the DSC of 0.8366, 0.8398 and 0.8100 for the RC, ZC, and GrabCut models. The result of our model (DSC: 0.9031) suggests that the introduction of the 3D curvature has the potential to maintain a proper balance between metric and geometric constraints.

**Fig. 10:**
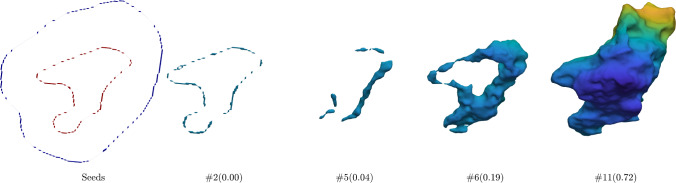
Intermediate contour evolution of the Ricci model for the post-resection cavity segmentation on the RESECT dataset. The final result is shown in Figure 11

Table 1 presents the metrics for these two organs on six scans. Our model achieves the highest IoU, HD, and DSC. Notably, SUNETR reaches a high HD of 67.4389 for the stomach segmentation because HD is sensitive to noise. For example, if a single additional voxel near the image boundary is added to the segmentation result, the DSC and IoU will barely change, but the HD will increase significantly. Due to the regularization, our model, RC, ZC, and GrabCut models can somewhat mitigate noise. SUNETR does not show a high DSC and IoU. This might be explained by the fact that, in contrast to the original paper that uses five-fold cross-validation to train the model, we only use the first fold, hence the model is trained only once.

The Ricci model exhibits considerable advantages across all metrics compared to the RC model, with 0.1038 IoU, 2.8756 HD, 0.0782 DSC improvement for the Esophagus segmentation and 0.1168 IoU, 4.814 HD, 0.0765 DSC improvement for the stomach segmentation. This might be due to the fact that the gradient relates curvature to intensity contrast. A high contrast, however, may not indicate a large intrinsic curvature. For example, at $(x,y)=(0,\,1)$, the unit circle’s derivative with respect to *x* is 0, and $\infty$ with respect to *y*. The curvature of a circle is, however, constant. This example demonstrates that the gradient depends on the choice of the coordinate system. By contrast, Ricci curvature is invariant under coordinate transformations, as discussed in Section 3.1.

### RESECT Dataset

We now focus on the post-resection cavity segmentation on four cases of the REtroSpective Evaluation of Cerebral Tumors (RESECT) dataset.

The RESECT dataset is used in the MICCAI CuRIOUS 2022 Segmentation Challenge (Xiao et al., 2017) aiming to track the intra-operative tissue shift and surgical tools for brain tumours and resection cavity at three surgical stages: pre-resection, during-resection, and post-resection. The three 3D US images come with annotations: the pre-resection brain tumour, the during-resection cavity, and the post-resection cavity. In addition, the three images reveal distinct brain anatomies and thus have different settings of shape (size), origin, and spacing. We only utilize the post-resection US image to segment the post-resection cavity. This is a common procedure as many studies consider the three segmentation tasks independently (Carton et al., 2020).

**Fig. 11:**
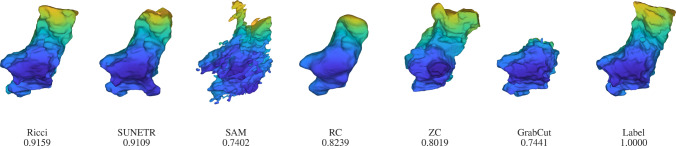
Segmentation results of all models and the ground truth for the case from Figure 10. (The SAM result was post-processed as before.)

The RESECT dataset comprises only 23 cases, and thus, we also fine-tuned a self-supervised pre-trained SUNETR model, which was identical to that of the BTCV dataset. We split the scans into 19 training scans and four validation scans. We first applied a sequence of data augmentation on the training images. Each 3D image was resampled with a voxel spacing of $0.2\times 0.2\times 0.2$ and randomly cropped to the size $96\times 96\times 96$, which also underwent random flips, rotations by 90 degrees, scalings, and intensity shifts. During training, we worked with a batch size of four. For optimization, we used Adam with decoupled weight decay employing a weighted sum of the Dice and cross-entropy loss. Finally, we validated all the models on the four validation scans. During validation, we only made an inference on the image region whose intensity was greater than zero. This can be viewed as a form of data augmentation cropping the foreground.

For the SAM model, we cropped all four scans to $128\times 128 \times 128$ and did not scale intensities as they were already in the $[0,\, 255]$ range. We segmented the whole 3D image slice by slice; that is, for each slice, we fed three consecutive slices into the SAM model: the previous slice, the current slice, and the next slice. We randomly selected three points in the foreground and 20 points in the background. Finally, we adopted the post-processing as mentioned before.

The configurations of the ZC and RC models remained the same as for the BTCV model since they are both single-channel 3D images. The iteration number for our model was set to 35, slightly higher than for the BTCV dataset, because the region of interest approximately spans half of the entire 3D image.

Figure 10 shows, for one example, the seed setting and the contour evolution of our proposed Ricci model. The two seed curves in the left-hand side figure are located in one slice. It is worth mentioning that it is not essential to enforce the foreground/background seeds to reside in the foreground/background of the ground truth because the distance term essentially provides a considerably rough segmentation. For example, the corresponding distance term ${\mathcal {D}}_G>0$, in this case, reaches a DSC of 0.2301.

Figure 11 shows the results of all models and their respective DSC. Our proposed model exhibits the best DSC of 0.9159, with an improvement of 0.005, 0.092, and 0.0114 over the SUNETR, RC, and ZC models, respectively. The SAM and GrabCut models achieve a similar DSC of around 0.74, possibly due to the limited intensity information of the image, which is approximately concentrated in the range $[0,\,60]$.

Table 1 summarizes the results across the three metrics. Our model reaches the highest values in IoU (0.8304), HD (15.5260), and DSC (0.9067). Remarkably, most models show high HD compared to the previous datasets. This is likely because the post-resection cavity sometimes accounts for about half of the whole 3D image, and a small displacement from the ground truth may produce a large HD.

The Ricci model demonstrates an improvement over the RC model of 0.1395 in IoU, 9.0695 in HD and 0.0903 in DSC. This might be explained by the low image contrast, which is mainly in the range of $[0,\,60].$ As mentioned before, this is hard to achieve using only the first-order gradient information. In addition, the relaxed level set’s histogram is mainly concentrated on the two ends of the interval $[0,\,1]$, implying that the preimage of 0 and 1 are 3D manifolds, which should be optimized through 3D geometric quantities, such as our proposed 3D Ricci curvature. By contrast, the traditional regularization assumes that all contours are 2D. For example, the variational calculus of $\int |\nabla u|\,\textrm{d}\Omega$ is $\nabla \cdot (\frac{\nabla u}{|\nabla u|})$, which may result in a division by zero in regions where $u=0$ or $u=1$.

**Fig. 12:**
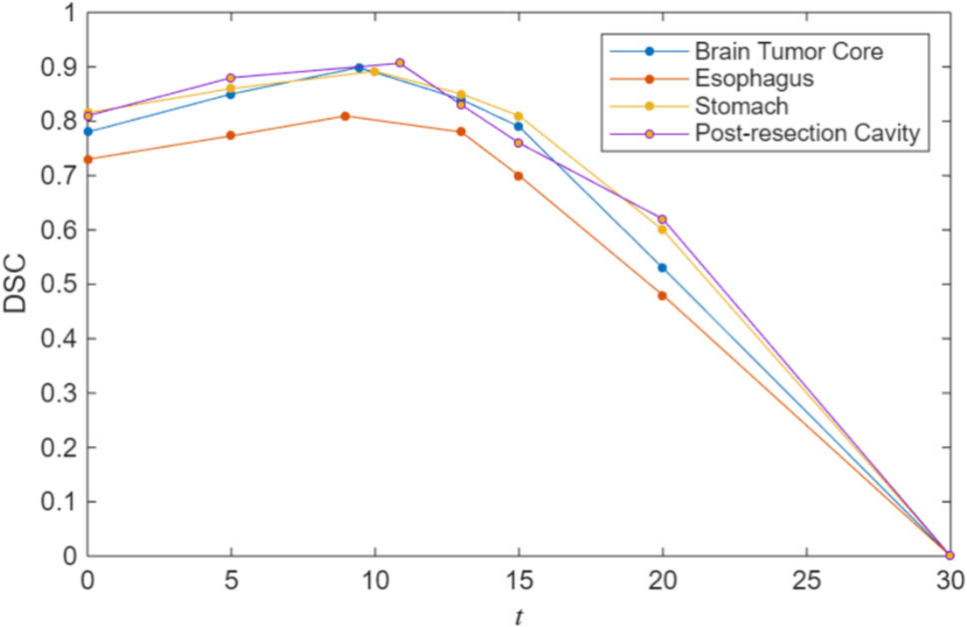
Ablation study to select the optimal *t* parameter in (21). The plot shows the average DSC metric for *t* ranging from 0 to 30 across three datasets. It can be seen that setting *t* to around 10 provides the highest DSC. The model fails to segment the image when $t=30$ because some intermediate functions reach infinity at that value, likely due to the exponential explosion in (21)

### Optimal *t* Parameter

We conclude the experiment by demonstrating the optimal time parameter *t* in (21). The *x* axis shows different values of *t* in the range $[0,\,30]$, while the *y* axis represents the average DSC metric for the four organs across the three datasets. When $t=0$, the model does not smooth the gradient field, resulting in a similar overall outcome to the RC model. As *t* increases, segmentation performance benefits from the Ricci curvature tensor derived from the metric tensor of the current manifold, leading to a rise in the DSC curve. It can be observed that the four curves peak at around $t=10$ and then show a faster DSC decrease compared to smaller *t* values. A possible reason is that the only parameter in (21) is *t*, while the scalar curvature tensor, metric tensor, and Ricci curvature tensor are fixed functions obtained from the last iteration. If *t* increases significantly, the exponential function may reach a very high value at some voxels, resulting in model instability. This phenomenon is observed in the DSC of 0 when $t=30$, where some infinity values appear in our Matlab implementation.

## Discussion and Conclusion

This paper introduces the 3D Ricci curvature tensor as regularization. Since the 3D image is considered a 3D hypersurface in Euclidean 4D space, we can derive the Euler-Lagrange equation with respect to the gradient instead of the routinely used metric tensor. Subsequently, we recover the original 3D function from the updated gradient.

Compared to the traditional models, the proposed model has two conceptually major virtues. First, as discussed in Section 3.1, our proposed functional (2) is invariant under coordinate transformations. That is, although we express it in a particular coordinate system, it is, in fact, independent of the choice of coordinates. By contrast, the traditional gradient-based regularization $\int |\nabla u|\,\textrm{d}\Omega$ may vary with the choice of the coordinate system. Second, as the relaxed level set is intended to approximate the Heaviside function, a step-shaped binary function whose isosurfaces are typically not 2D surfaces, it seems more natural to consider 3D manifolds, as in our approach, instead of sequences of 2D manifolds.

Moreover, curvature-based regularization may be beneficial in cases where gradient-based regularization runs into numerical problems, for instance, due to division by zero in the variational calculus.

We believe that 3D intrinsic curvatures as regularization is a fruitful area for further research. In practice, the manifold structure in specific applications is not arbitrary. For instance, in the present paper, we have modelled the manifold as a hypersurface. In more specific cases, it may be possible to directly derive the variational calculus of the regularization relative to its gradient. If the structure can be further restricted to a low-dimensional parameter space (e.g., the radius for a sphere), it may be possible to directly derive the updating strategy with respect to these parameters. In other words, although the form of variational calculus may vary with the specific setting of the 3D manifold structure, it may be possible to derive the exclusive updating strategy tailored to the assumed specific configuration. An analogous approach may be applicable to a wider range of curvature flows of the form $\frac{\partial {\textbf{g}}}{\partial t}=\kappa$, where $\kappa$ is an intrinsic curvature, such as the renowned Ricci flow (Milnor, 2003).

Another interesting direction for further research may be to incorporate curvature flow into a deep learning framework. We note that Euler’s Elastica has been introduced into the loss function, such as in Chen et al. (2021), to improve image segmentation. We plan to incorporate 3D curvature into the loss function to enhance 3D image segmentation. However, the straightforward use of the high-order loss function may not show promising results because some boundary terms (e.g., the divergence appearing in (15)) involved in the variational calculus may vanish. Thus, the backpropagation for the curvature-based loss function needs to be adapted.

## Data Availability

The BraTS 2021 dataset is available from the Cancer Imaging Archive: https://www.cancerimagingarchive.net/analysis-result/rsna-asnr-miccai-brats-2021/. The Beyond the Cranial Vault (BTCV) Segmentation Challenge dataset is available from Synapse: https://www.synapse.org/Synapse:syn3193805/wiki/217789. The REtroSpective Evaluation of Cerebral Tumors (RESECT) dataset is available from the Nird Research Data Archive: https://archive.norstore.no/pages/public/datasetDetail.jsf?id=10.11582/2017.00004.

## Riemann Curvature Tensor of 3D Images

In this section, we derive the Riemann curvature tensor $R_{ijkl}$, a $3\times 3\times 3\times 3$ matrix, for the 3D parametric surface ${\textbf{r}}\left( x,\,y,\,z\right) =(x,\,y,\,z,\,u(x,\,y,\,z))$.

For each point (*x*, *y*, *z*) on ${\textbf{r}}$, in addition to the three tangents, ${\textbf{r}}_1=(1,\,0,\,0,\,u_x)$, ${\textbf{r}}_2=(0,\,1,\,0,\,u_y)$, and ${\textbf{r}}_3=(0,\,0,\,1,\,u_z)$, there exists a unit vector ${\textbf{n}}=\frac{(u_x,\,u_y,\,u_z,\,-1)}{\sqrt{1+u_x^2+u_y^2+u_z^2}}$ that is perpendicular to the three vectors.

With these four vectors, the Riemann curvature tensor $R_{ijkl}$ can be defined according to the Gauss equation (Dajczer & Tojeiro, 2019) as follows:

A1 $$\begin{aligned} \begin{aligned} R_{ijkl} =&\mathrm {I\!I}({\textbf{r}}_i,{\textbf{r}}_k)\mathrm {I\!I} ({\textbf{r}}_j,{\textbf{r}}_l)-\mathrm {I\!I} ({\textbf{r}}_i,{\textbf{r}}_l)\mathrm {I\!I} ({\textbf{r}}_j,{\textbf{r}}_k), \end{aligned} \end{aligned}$$

where $\mathrm {I\!I}({\textbf{r}}_i,{\textbf{r}}_j)$ denotes the second fundamental form, defined by the projection of the second-order partial derivative ${\textbf{r}}_{ij}$ onto the normal vector ${\textbf{n}}$ of the surface:

$$\begin{aligned} \mathrm {I\!I}({\textbf{r}}_i,{\textbf{r}}_j)=<{\textbf{r}}_{ij},{\textbf{n}}>. \end{aligned}$$

By the usual second-order partial derivative ${\textbf{r}}_{ij}=(0,\,0,\,0,u_{ij})$, the second fundamental is given by:

A2 $$\begin{aligned} \mathrm {I\!I}({\textbf{r}}_i,{\textbf{r}}_j)=\frac{-u_{ij}}{\sqrt{1+u_x^2+u_y^2+u_z^2}}. \end{aligned}$$

Substituting the above formula into (A1), we obtain:

A3 $$\begin{aligned} \begin{aligned} R_{ijkl} =\frac{u_{ik}u_{jl}-u_{il}u_{jk}}{1+{|\nabla u|}^2}. \end{aligned} \end{aligned}$$

## Transformation Invariance of the Einstein-Hilbert Action

Assume the original point $(x,\,y,\,z)$ has the metric tensor ${\textbf{g}}$ (3) and the second fundamental form denoted by $\mathrm {I\!I}$ (A2). Under a coordinate transformation with the Jacobian matrix *J*, the new point with coordinates $({\tilde{x}}(x,\,y,\,z),\,{\tilde{y}}(x,\,y,\,z),\,{\tilde{z}}(x,\,y,z))$ has the metric tensor $\tilde{{\textbf{g}}}$ and the second fundamental form $\tilde{\mathrm {I\!I}}$. Recall that the metric tensor and the second fundamental form are bilinear forms that map two tangent vectors into a real number. Since the Jacobian matrix *J* is a linear map from the original tangent space to the transformed tangent space, it follows from linear algebra that the two bilinear forms have the following relationships:

$$\begin{aligned} {\textbf{g}}&={\textbf{J}}^{\operatorname {T}}\tilde{{\textbf{g}}}{\textbf{J}}\\ \mathrm {I\!I}&={\textbf{J}}^{\operatorname {T}}\tilde{\mathrm {I\!I}}{\textbf{J}}. \end{aligned}$$

It is easy to see that $\operatorname {det}({\textbf{g}})=1+{|\nabla u|}^2$, and according to the definition of the Einstein-Hilbert Action (2), we have

$$\begin{aligned}&\phantom {=}\frac{{\left( \operatorname {tr}\left( {\textbf{g}}^{-1}{\textbf{H}}\right) \right) }^2-\operatorname {tr}\left( {\textbf{g}}^{-1}{\textbf{H}}{\textbf{g}}^{-1}{\textbf{H}}\right) }{\sqrt{1+{|\nabla u|}^2}}\,\textrm{d}x\textrm{d}y\textrm{d}z\\&\quad =\frac{{\left( \operatorname {tr}\left( {\textbf{g}}^{-1}{\textbf{H}}\right) \right) }^2- \operatorname {tr}\left( {\textbf{g}}^{-1}{\textbf{H}}{\textbf{g}}^{-1}{\textbf{H}}\right) }{\operatorname {det}{{\textbf{g}}}}\\&\quad \sqrt{\operatorname {det}{{\textbf{g}}}}\,\textrm{d}x\textrm{d}y\textrm{d}z\\&\quad =\left( {\left( \operatorname {tr}\left( {\textbf{g}}^{-1}\frac{{\textbf{H}}}{\sqrt{\operatorname {det}{{\textbf{g}}}}}\right) \right) }^2\right. \\&\quad \left. -\operatorname {tr}\left( {\textbf{g}}^{-1}\frac{{\textbf{H}}}{\sqrt{\operatorname {det}({\textbf{g}})}}{\textbf{g}}^{-1}\frac{{\textbf{H}}}{\sqrt{\operatorname {det}({\textbf{g}})}}\right) \right) \\&\quad \sqrt{\operatorname {det}{{\textbf{g}}}}\,\textrm{d}x\textrm{d}y\textrm{d}z\\&\quad =\left( {\left( \operatorname {tr}\left( {\textbf{g}}^{-1}\mathrm {I\!I}\right) \right) }^2-\operatorname {tr}\left( {\textbf{g}}^{-1}\mathrm {I\!I}{\textbf{g}}^{-1}\mathrm {I\!I}\right) \right) \sqrt{\operatorname {det}{{\textbf{g}}}}\,\textrm{d}x\textrm{d}y\textrm{d}z \end{aligned}$$

Then we express the above formula using $\tilde{{\textbf{g}}}$ and $\tilde{\mathbf {\mathrm {I\!I}}}$ as:

$$\begin{aligned}&\phantom {=}\left( {\left( \operatorname {tr}\left( {\textbf{g}}^{-1}\mathrm {I\!I}\right) \right) }^2-\operatorname {tr}\left( {\textbf{g}}^{-1}\mathrm {I\!I}{\textbf{g}}^{-1}\mathrm {I\!I}\right) \right) \sqrt{\operatorname {det}{{\textbf{g}}}}\,\textrm{d}x\textrm{d}y\textrm{d}z\\&\quad =\left( {\left( \operatorname {tr}\left( {\textbf{J}}^{-1}{\tilde{{\textbf{g}}}}^{-1}{\textbf{J}}^{-\operatorname {T}}{\textbf{J}}^{\operatorname {T}}\tilde{\mathrm {I\!I}}{\textbf{J}}\right) \right) }^2\right. \\&\quad -\left( \operatorname {tr}\left( {\textbf{J}}^{-1}{\tilde{{\textbf{g}}}}^{-1}{\textbf{J}}^{-\operatorname {T}}{\textbf{J}}^{\operatorname {T}}\tilde{\mathrm {I\!I}}{\textbf{J}}{\textbf{J}}^{-1}{\tilde{{\textbf{g}}}}^{-1}{\textbf{J}}^{-\operatorname {T}}{\textbf{J}}^{\operatorname {T}}\tilde{\mathrm {I\!I}}{\textbf{J}}\right) \right) \\&\quad \sqrt{\operatorname {det}{\left( {\textbf{J}}^{\operatorname {T}}\tilde{{\textbf{g}}}{\textbf{J}}\right) }}\operatorname {det}({{\textbf{J}}}^{-1})\,\textrm{d}{\tilde{x}}\textrm{d}{\tilde{y}}\textrm{d}{\tilde{z}}\\&\quad =\left( {\left( \operatorname {tr}\left( {\textbf{J}}^{-1}{\tilde{{\textbf{g}}}}^{-1}\tilde{\mathrm {I\!I}}{\textbf{J}}\right) \right) }^2-\operatorname {tr}\left( {\textbf{J}}^{-1}{\tilde{{\textbf{g}}}}^{-1}\tilde{\mathrm {I\!I}}{\tilde{{\textbf{g}}}}^{-1}\tilde{\mathrm {I\!I}}{\textbf{J}}\right) \right) \\&\quad \sqrt{\operatorname {det}{\left( {\textbf{J}}^{\operatorname {T}}\tilde{{\textbf{g}}}{\textbf{J}}\right) }}\operatorname {det}({{\textbf{J}}}^{-1})\,\textrm{d}{\tilde{x}}\textrm{d}{\tilde{y}}\textrm{d}{\tilde{z}}. \end{aligned}$$

Since $\operatorname {tr}(\textbf{ABC})=\operatorname {tr}(\textbf{BCA})$ and $\operatorname {det}(\textbf{AB})=\operatorname {det}({\textbf{A}})\operatorname {det}({\textbf{B}})$, we obtain

$$\begin{aligned}&\phantom {=}\left( {\left( \operatorname {tr}\left( {\textbf{g}}^{-1}\mathrm {I\!I}\right) \right) }^2-\operatorname {tr}\left( {\textbf{g}}^{-1}\mathrm {I\!I}{\textbf{g}}^{-1}\mathrm {I\!I}\right) \right) \sqrt{\operatorname {det}{{\textbf{g}}}}\,\textrm{d}x\textrm{d}y\textrm{d}z\\&\quad =\Big ({\big (\operatorname {tr}\big ({\tilde{{\textbf{g}}}}^{-1}\tilde{\mathrm {I\!I}}\big )\big )}^2-\operatorname {tr}\big ({\tilde{{\textbf{g}}}}^{-1}\tilde{\mathrm {I\!I}}{\tilde{{\textbf{g}}}}^{-1}\tilde{\mathrm {I\!I}}\big )\Big )\sqrt{\operatorname {det}{\tilde{{\textbf{g}}}}}\,\textrm{d}{\tilde{x}}\textrm{d}{\tilde{y}}\textrm{d}{\tilde{z}}. \end{aligned}$$

## Derivation of (16)

Following the convention in Section 3.3, all tensors are functions of the gradient. From (A3) and (5), we see that

C4 $$\begin{aligned}&R_{ql}=g^{sk}R_{sqkl}=\frac{g^{sk}\left( v_{sk}v_{ql}-v_{sl}v_{qk}\right) }{1+{|{\textbf{v}}|}^2} \end{aligned}$$

From the Sherman-Morrison formula (Petersen & Pedersen, 2008), it is straightforward to see that the inverse matrix ${\textbf{g}}^{-1}$ is

$$\begin{aligned} {\textbf{g}}^{-1}={\textbf{I}}-\frac{{\textbf{v}} {{\textbf{v}}}^{\operatorname {T}}}{1+{|{\textbf{v}}|}^2}, \end{aligned}$$

and thus,

$$\begin{aligned} {{\textbf{v}}}^{\operatorname {T}}{\textbf{g}}^{-1}{\textbf{v}}={|{\textbf{v}}|}^2-\frac{{|{\textbf{v}}|}^4}{1+{|{\textbf{v}}|}^2}=\frac{{|{\textbf{v}}|}^2}{1+{|{\textbf{v}}|}^2}, \end{aligned}$$

yielding

$$ \frac{1}{1+{|{\textbf{v}}|}^2}=1- {{\textbf{v}}}^{\operatorname {T}}{\textbf{g}}^{-1}{\textbf{v}}=1-g^{ab}v_av_b. $$

After displacing the term $\frac{1}{1+{|{\textbf{v}}|}^2}$, (C4) is written as

$$ \begin{aligned}&R_{ql}=g^{sk}\left( v_{sk}v_{ql}-v_{sl}v_{qk}\right) \left( 1-g^{ab}v_av_b\right) \end{aligned} $$

Observe that $v_{sk}v_{ql}-v_{sl}v_{qk}=\frac{\partial v_sv_{ql}}{\partial k}-\frac{\partial v_sv_{qk}}{\partial l}$, and we substitute into the above equation

$$\begin{aligned}&R_{ql}= g^{sk}\left( v_{sk}v_{ql}-v_{sl}v_{qk}\right) \\&\quad -g^{sk}\left( v_{sk}v_{ql}-v_{sl}v_{qk}\right) g^{ab}v_av_b\\&\quad = g^{sk}\left( \frac{\partial v_sv_{ql}}{\partial k}-\frac{\partial v_sv_{qk}}{\partial l}\right) \\&\quad -g^{sk}\left( v_{sk}v_{ql}-v_{sl}v_{qk}\right) g^{ab}v_av_b\\&\quad =g^{sk}\frac{\partial v_sv_{ql}}{\partial k}-g^{sk}v_{sk}v_{ql}g^{ab}v_av_b\\&\quad -g^{sk}\frac{\partial v_sv_{qk}}{\partial l}+g^{sk}v_{sl}v_{qk}g^{ab}v_av_b\\&\quad =\frac{\partial g^{sk}v_sv_{ql}}{\partial k}-v_sv_{ql}\frac{\partial g^{sk}}{\partial k}-g^{sk}v_{sk}v_{ql}g^{ab}v_av_b\\&\quad -\frac{\partial g^{sk}v_sv_{qk}}{\partial l}+v_sv_{qk}\frac{\partial g^{sk}}{\partial l}+g^{sk}v_{sl}v_{qk}g^{ab}v_av_b\\ \end{aligned}$$

From the fact that $\frac{\partial g^{sk}}{\partial k}=-g^{sa}g^{kb}v_{ak}v_b-g^{sa}g^{kb}v_{a}v_{bk}$ and $\frac{\partial g^{sk}}{\partial l}=-g^{sa}g^{kb}v_{al}v_b-g^{sa}g^{kb}v_{a}v_{bl}$, it follows that

$$\begin{aligned}&R_{ql}=\frac{\partial g^{sk}v_sv_{ql}}{\partial k}+v_sv_{ql}g^{sa}g^{kb}v_{ak}v_b\\&\quad v_sv_{ql}g^{sa}g^{kb}v_{a}v_{bk}-g^{sk}v_{sk}v_{ql}g^{ab}v_av_b\\&\quad -\frac{\partial g^{sk}v_sv_{qk}}{\partial l}-v_sv_{qk}g^{sa}g^{kb}v_{al}v_b\\&\quad -v_sv_{qk}g^{sa}g^{kb}v_{a}v_{bl}+g^{sk}v_{sl}v_{qk}g^{ab}v_av_b \end{aligned}$$

According to the Einstein summation convention, if both indices *i* and *j* appear twice, they can be interchanged. Thus, $v_sv_{ql}g^{sa}g^{kb}v_{a}v_{bk}=g^{sk}v_{sk}v_{ql}g^{ab}v_av_b$ and $v_sv_{qk}g^{sa}g^{kb}v_{a}v_{bl}=g^{sk}v_{sl}v_{qk}g^{ab}v_av_b$. At this point, four of the six terms from the above formula vanish, yielding

$$\begin{aligned}&R_{ql}=\frac{\partial g^{sk}v_sv_{ql}}{\partial k}+v_sv_{ql}g^{sa}g^{kb}v_{ak}v_b\\&\quad -\frac{\partial g^{sk}v_sv_{qk}}{\partial l}-v_sv_{qk}g^{sa}g^{kb}v_{al}v_b \end{aligned}$$
